# Increased Serum Neurofilament Light Chain Concentration Associated With Microglial Morphology Changes in Chronically‐Starved Mice

**DOI:** 10.1002/eat.24423

**Published:** 2025-03-22

**Authors:** Annelie Zimmermann, Hanna Rupprecht, Stephan Lang, Rickmer Wienecke, Hanna‐Sophia Henschke, Kaja Dickert, Katharina Schuster, Anna Staffeld, Christoph Berger, Alexander Dück, Michael Kölch, Antje Vogelgesang, Matthias Grothe, Leo Heinig, Lukas Wenzel, Markus Kipp, Linda Frintrop

**Affiliations:** ^1^ Institute of Anatomy Rostock University Medical Center Rostock Germany; ^2^ Department of Psychiatry, Neurology, Psychosomatics, and Psychotherapy in Childhood and Adolescence Rostock University Medical Center Rostock Germany; ^3^ German Center for Child and Adolescent Health (DZKJ), Partner Site Greifswald/Rostock Rostock Germany; ^4^ Department of Neurology University Medicine Greifswald Greifswald Germany

**Keywords:** anorexia nervosa, brain volume, microglia, neurofilament light chain, neurolucida, neuronal damage, refeeding, starvation‐induced hyperactivity mouse model

## Abstract

**Objective:**

Anorexia nervosa (AN) is associated with hyperactivity, amenorrhea, and brain atrophy. Weight rehabilitation reversed these symptoms, although the underlying pathophysiological mechanisms are mostly unknown. Serum neurofilament light chain (NfL) levels are widely used as a biomarker of neurodegeneration. Based on neuroimaging studies and increased serum NfL levels, we assume that neurodegeneration is a core neuropathological feature in AN patients.

**Method:**

Female mice were given a limited amount of food once a day and had unlimited access to a running wheel until they reached a 25% weight reduction, which was maintained for 2 weeks to mimic chronic starvation. This was followed by 3 weeks of refeeding. Running activity was measured by wheel sensors, while amenorrhea was determined by analyzing vaginal smears. Brain sections were used to investigate brain volumes. NfL levels were determined using a NF‐light assay. Behavioral tests such as forced swim and elevated plus maze assessed behavioral changes. Immunohistochemistry was used to quantify the density of microglia, while their morphological analysis was performed using Neurolucida 360.

**Results:**

Chronic starvation led to AN‐related symptoms of hyperactivity and amenorrhea. The decreased cerebral cortex, hippocampal, and corpus callosum volumes were paralleled by increased NfL levels after chronic starvation. A behavioral association was reduced anxiety‐like behavior after chronic starvation. Starvation induced decreased microglial density, increased soma area, and prolonged microglial processes.

**Discussion:**

Chronic starvation led to an increase in NfL levels and changed microglial morphology in a mouse model of AN, suggesting that neuronal pathophysiology may contribute to the disease.

**Summary**

- Anorexia nervosa (AN) is associated with brain atrophy, but the underlying mechanisms remain poorly understood.
- This study used a mouse model mimicking AN to explore the effects of starvation on the brain.
- The findings revealed that starvation induces brain volume loss, elevates a biomarker of neurodegeneration, and alters microglial cell density and morphology, indicating that neuronal dysfunction may play a role in the pathology of AN.

## 1 Introduction

Anorexia nervosa (AN) is an eating disorder characterized by severe weight loss due to a body image disturbance with a frequently chronic course (Moskowitz and Weiselberg 2017; Herpertz‐Dahlmann 2015). In addition, AN, for which treatment options are limited, is associated with hyperactivity, amenorrhea (Herpertz‐Dahlmann 2015), and an elevated mortality rate in later life (van Eeden et al. 2021). Neuroimaging studies, including those conducted by the worldwide ENIGMA consortium, in individuals with AN have demonstrated significant reductions in both gray and white matter brain volumes (Seitz et al. 2014; Walton et al. 2022; Tose et al. 2024). However, it remains unclear to what extent this brain atrophy is reversible.

The cellular mechanisms underlying brain atrophy are largely unknown, as only a few post‐mortem case studies have analyzed the brains of AN patients, with data suggesting altered neuronal cell numbers, neuronal morphology, and astrogliosis (Neumärker et al. 1997; Martin 1958; Kawakami et al. 2022). In addition to these structural alterations, functional magnetic resonance imaging (fMRI) studies indicate altered neural activity across the entire brain. For example, fMRI studies including neuropsychological tasks have shown reduced activity of the prefrontal cortex and thalamus (Fuglset et al. 2016). Moreover, a fMRI study involving a working memory task in adolescent AN patients revealed an association between gray matter volume loss and impaired visuospatial memory (Castro‐Fornieles et al. 2010). A further study reported that brain atrophy is positively correlated with impaired logical thinking (McCormick et al. 2008). Furthermore, depression and anxiety disorders frequently co‐occur with AN, representing the most prevalent comorbid conditions in AN (Salbach‐Andrae et al. 2008). However, the underlying mechanisms of neuropsychological deficits in AN patients remain unclear.

A limited number of animal models are available to study the cellular mechanisms underlying AN, with the activity‐based anorexia (ABA) model being historically the most widely utilized. The ABA model combines food restriction for a few hours per day (1–3 h) with running wheel access. Although this model mimics AN‐related symptoms, such as weight loss and hyperactivity, the excessive activity observed during the feeding periods has led to an increased risk of mortality (Routtenberg and Kuznesof 1967). To mitigate the risk of mortality, we developed a modified AN model called the starvation‐induced hyperactivity (SIH) model. This approach includes providing an individually calculated, restricted amount of food once daily, along with access to a running wheel (Staffeld et al. 2023; Frintrop et al. 2018). Compared to the dehydration‐induced anorexia (DIA) model, which combines drinking hypertonic saline and free access to food (Watts and Boyle 2010; Reyes‐Haro et al. 2016) and another model that makes use of mice homozygous for the anorexia (*anx*) mutation (Maltais et al. 1984), the advantage of the SIH model is the possibility to induce chronic starvation, thus recapitulating the chronic course of AN.

Our prior research has shown that the SIH model successfully replicates key somatic features of AN, including body weight loss, amenorrhea, and hyperactivity (Staffeld et al. 2023; Frintrop et al. 2018). Furthermore, we recently demonstrated that chronic starvation leads to a decreased density of oligodendrocytes, astrocytes, and microglia in the corpus callosum (Zimmermann et al. 2023). Additionally, our previous studies demonstrated that starvation‐induced changes in rats, including reduced astrocyte density and brain atrophy, were largely reversed following refeeding (Frintrop et al. 2019). Furthermore, chronic starvation in rats led to impaired recognition memory (Paulukat et al. 2016). However, possible associations between behavioral changes of AN and brain atrophy remain less studied.

A well‐known clinical biomarker of neurodegenerative diseases is the neurofilament light chain (NfL), which is exclusively expressed in neurons and highly specific for neuronal cell damage (Khalil et al. 2018). Since NfL can be measured in cerebrospinal fluid and serum, its levels have been studied in several neurological diseases, including multiple sclerosis, amyotrophic lateral sclerosis, and Parkinson's disease (Gaetani et al. 2019). Furthermore, studies in AN patients showed increased serum levels of NfL (Nilsson et al. 2019; Hellerhoff et al. 2021). We used the reverse translational approach of the SIH model using NfL as a valuable biomarker of neurodegenerative disease (Loeffler et al. 2020; Bacioglu et al. 2016). This may reveal whether the brain atrophy observed in AN is associated with neuronal damage. In addition, microglial cell density and morphology were analyzed as an indirect marker of neuronal damage, as neuronal dysfunction is associated with changes in both. Moreover, the SIH model with a refeeding phase provides insights into whether neuronal damage is ongoing after weight recovery.

In this study, we aimed to investigate whether starvation and concomitant hyperactivity result in neurodegeneration. In parallel, behavioral tests were used to determine whether neuronal dysfunction is paralleled by behavioral abnormalities.

## 2 Material and Methods

### 2.1 Animals

Female C57BL/6J mice (4 weeks old), obtained from Janvier Labs (Le Genest‐Saint‐Isle, France) were selected due to the higher prevalence of AN in females (Galmiche et al. 2019; Jagielska and Kacperska 2017; Keski‐Rahkonen and Mustelin 2016; Silén and Keski‐Rahkonen 2022). Accordingly, amenorrhea as a symptom of AN indicates a sufficient level of starvation (Herpertz‐Dahlmann 2015).

All mice were housed individually in a cage under a 12/12 h light/dark cycle (lights on at 6 AM) at a temperature of 23°C ± 2°C. During the whole experiment, all mice had unlimited access to a running wheel. Furthermore, the cages were changed weekly, and microbiological monitoring was performed according to the Federation of European Laboratory Animal Science Associations (FELASA) recommendations. The animal studies were approved by the Review Boards for the Care of Animal Subjects of the district government of Mecklenburg‐Western Pomerania (reference number 7221.3‐1‐005/21).

### 2.2 Study Design

**FIGURE 1:**
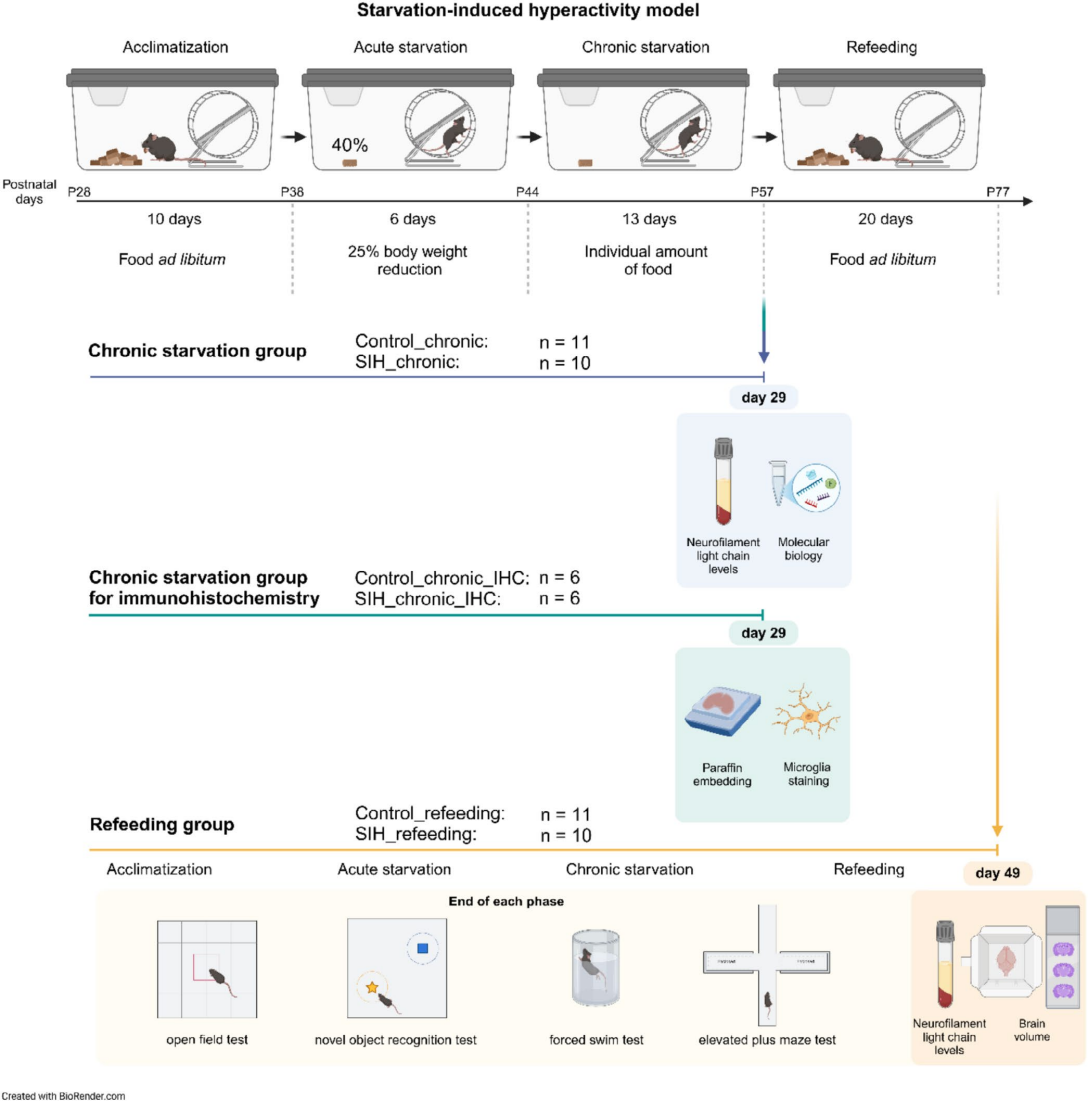
Schematic summary of the experimental set‐up, including various cohorts used in the present study. Created with BioRender.com.

**FIGURE 2:**
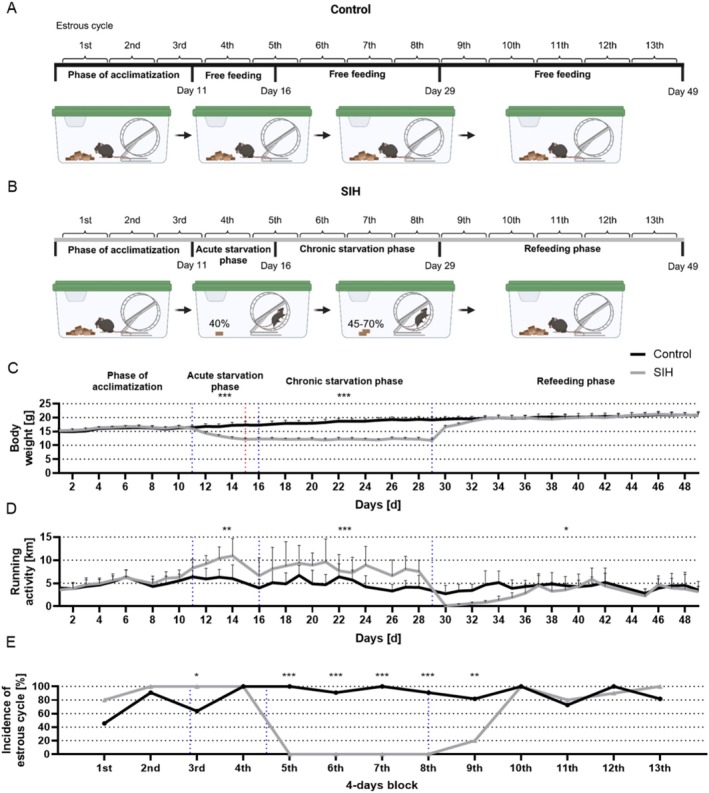
Chronic starvation in SIH mice leads to AN‐related symptoms such as hyperactivity and amenorrhea. (A, B) A schematic experimental set‐up of refeeding after chronic starvation with SIH and control mice is presented. Created with BioRender.com. (C) The body weight, (D) running wheel activity, and (E) incidence of estrous cycle within 4‐days blocks determined by histological analyses of vaginal smears were measured daily at 1 PM. Blue dotted lines represent the start of acute starvation, chronic starvation, or refeeding. The red dotted line highlights the time point when the SIH group reached a 25% body weight loss. (C, D) Two‐way ANOVA with repeated measurements was performed to compare body weight and running wheel activity between SIH and control mice for the different phases (phase of acclimatization, acute starvation phase, chronic starvation phase, or refeeding phase). (E) The incidence of mice with estrous was analyzed using Chi‐square tests comparing SIH and control mice for each block separately, *p ≤ 0.05, **p ≤ 0.01, ***p ≤ 0.001.

Starvation‐induced hyperactivity was induced as published previously by our group (Frintrop et al. 2018). Figure 1 presents a schematic structure of the SIH paradigm with refeeding, including the corresponding investigations of the different animal cohorts. At the start of the experiment, the mice underwent a 10‐day acclimatization phase with unlimited access to food and water. Body weight, food intake, and menstrual cycle were determined daily at 1 PM. After this acclimatization phase, the mice were randomly assigned to the different experimental groups (Figure 2A,B, Control_chronic: *n* = 11; SIH_chronic: *n* = 10; Control_refeeding: *n* = 11; SIH_refeeding: *n* = 10; 4 weeks old referred to as juvenile; one animal from each SIH group was excluded due to one of termination criteria). The acute starvation phase was defined as a 6 days phase, in which the SIH mice received 40% of the baseline food intake (provided at 1 PM), calculated from the averaged food intake during the acclimatization phase until a 25% body weight reduction was obtained. To mimic chronic starvation, daily food intake was adjusted to sustain a 25% body weight reduction for an additional 2 weeks, corresponding to 45%–70% of the baseline food intake. During the acute and chronic starvation phases, SIH animals had unrestricted access to their food amount (Ssniff, Soest, Germany). After chronic starvation, mice were returned to food *ad libitum* in the refeeding phase which lasts 21 days. Control mice were fed *ad libitum* throughout the whole experiment. For immunohistochemical evaluation and morphological analysis, an additional animal study was performed using the same parameters (25% weight reduction, early adolescent mice (4 weeks old) and chronic starvation), leading to hyperactivity and amenorrhea during chronic starvation (Control_chronic_IHC: *n* = 6; SIH_chronic_IHC: *n* = 6). Running wheel activity and estrous cycle were analyzed as previously described in (Staffeld et al. 2023; Frintrop et al. 2018; Gabloffsky et al. 2022) (further details: Data S1). Mice were removed from the study in the case of termination criteria including a 10% additional weight loss within 24 h, the presence of cramps, paralysis, breathing noises, or forced breathing.

### 2.3 Tissue Preparation and Brain Volume Measurement

At the end of the experiment, the mice were injected with ketamine (100 mg/kg) and xylazine (10 mg/kg) and transcardially perfused using phosphate‐buffered saline followed by a 3.7% paraformaldehyde solution (pH 7.4). For brain volume measurements, standard tissue preparation procedures were followed, including the sectioning of brains into 40 μm frontal sections for Nissl staining (Staffeld et al. 2023; Zimmermann et al. 2023) (further details: Data S1). For immunohistochemistry, brains were embedded in paraffin and sectioned into 5 μm sections.

Brain volume analysis was performed on Nissl‐stained sections, with areas manually traced by a blinded observer using ImageJ software (Schneider et al. 2012) (version 1.48v, Wayne Rasband, National Institutes of Health, Bethesda, MD, USA) and volumes calculated via the Cavalieri method (further details: Data S1). Regions analyzed included the cerebral cortex, hippocampus, and corpus callosum, defined according to the Paxinos and Franklin Mouse Atlas (Franklin and Paxinos 2005). Brain volumes after refeeding (Control_refeeding: *n* = 11; SIH_refeeding: *n* = 10) were compared with those obtained in our previous study in chronically‐starved mice (Staffeld et al. 2023).

### 2.4 Measurement of Serum Neurofilament Light Chain

To evaluate serum NfL levels, blood samples were taken at the end of chronic starvation (Control_chronic: *n* = 9; SIH_chronic: *n* = 8) and after the refeeding phase (Control_refeeding: *n* = 9; SIH_refeeding: *n* = 7) via retro‐orbital puncture. To obtain serum, blood samples were centrifuged at 3400 g for 15 min at 4°C, and the supernatant was collected. NfL was quantified by Simoa NF‐light V2 Advantage Kit on a SR‐X Biomarker Detection System (both Quanterix Corp., Billerica, MA, USA) according to manufacturer instructions. Due to insufficient serum volumes, some animals were excluded from the analyses.

### 2.5 Immunohistochemistry and Image Analysis

For histological evaluation of microglia with the marker ionized calcium‐binding adapter molecule 1 (IBA1), paraffin‐embedded brain sections were processed following established protocols (Beecken et al. 2023) (further details: Data S1, Table S1). The anterior cingulate and secondary motor area of the cortex (region of interest, ROI) were outlined above the medial corpus callosum with a perpendicular line dropped from the brain surface and the peak of the cingulum as the lateral boundary according to (Wittekindt et al. 2022). Furthermore, Random Tree‐based pixel classifiers in QuPath were used to measure the area of microglial soma and processes within the ROI, reported as % of the ROI (further details: Data S1).

### 2.6 Morphological Analysis

For detailed morphological analysis, 89 microglial cells from the control_chronic_IHC (*n* = 4) and 65 microglial cells from SIH_chronic_IHC mice (*n* = 5) in the anterior cingulate and secondary motor area of the cortex were digitally reconstructed with Neurolucida 360 (MBF Bioscience, Willistion, USA; Version 2022.1.1, further details: Data S1). The following parameters were evaluated: soma area per cell, processes per cell, mean process length, nodes per cell, endpoints per cell, intersections within Sholl shell, and volume of processes within Sholl shell. Furthermore, resulting data were used to calculate a ramification index (RI), which is defined as the ratio of the cell area (*A*_*c*_) to the projection area (*A*_*p*_) (Kogel et al. 2021; Heppner et al. 1998). The RI distinguishes microglial activation states: resting microglia have small somas with long, thin processes. Activated microglia show hypertrophic somas and retracted processes, thus the RI approaches a value close to 1 (reviewed in (Reddaway et al. 2023)).

### 2.7 Behavioral Tests

For the behavioral analyses (further details: Data S1; Control_refeeding: *n* = 11; SIH_refeeding: *n* = 10), several tests were performed after the acclimatization phase, after acute starvation, after chronic starvation, and after refeeding.

The forced swim test (FST) was used to determine depressive‐like behavior (Porsolt et al. 1978, 1977). Mice were placed in a water‐filled cylinder, and the times of swimming and immobile floating were measured over 5 min. A mouse with increased depression‐like behavior spends more time immobile than swimming.

The elevated plus maze (EPM) and open field (OF) tests were used to determine anxiety‐like behavior based on the natural behavior of mice avoiding open or light areas (Hall 1934; Montgomery 1955). In the EPM, mice explored a setup with two open and two closed arms, and time spent in each was recorded automatically. A mouse with increased anxiety‐like behavior spends more time in the closed arms than in the open arms. In the OF test, mice were placed in an open box, with time spent at the edges and the center recorded automatically. A mouse with increased anxiety‐like behavior spends more time at the edges of the box compared to the center of the box.

The novel object recognition (NOR) test was used to analyze recognition memory by measuring the ability of mice to recognize familiar objects and was modified from previously described protocols (Paulukat et al. 2016; Luine and Frankfurt 2012; Lueptow 2017). After a training phase with identical objects, one object was replaced by a novel object. Recognition memory was assessed by the exploration index, calculated as the time spent on the novel object relative to both objects, with increased values indicating better recognition memory.

All animals were included in the results from FST, EPM, and OF test. In the NOR test, mice with an exploration time of less than 30 s were excluded to ensure sufficient exploration with the objects.

### 2.8 Statistics

The data are presented as means and standard deviations (SD). The Shapiro–Wilk test was used to test for normal distribution, and the Levene test was used to test for homogeneity of variance. All data and statistics can be found in Table S2. For the statistical analysis, the values for body weight and daily running activity of the different phases were compared: acclimatization phase (days 1–10), acute starvation phase (days 11–16), chronic starvation phase (days 17–29) and refeeding phase (days 30–49). The analysis of body weight and 24‐h running activity between SIH and control mice within each phase of starvation was performed using two‐way ANOVA with repeated measurements, with a significance level of 5%. Bonferroni correction was used for post hoc evaluations between the control and SIH groups. To investigate whether the estrous cycle differed between the control and SIH groups, the presence or absence of the fertile phase in each 4‐days block was determined. As this is a nominal scale level, the Chi‐square test was used. In addition, brain volumes, serum NfL levels, and behavioral tests were also analyzed with two‐way ANOVA followed by Bonferroni correction. Cell count and morphology data were analyzed using Student's *t*‐test for normally distributed data or the Mann–Whitney test for non‐normally distributed data. For Sholl analysis, the mixed‐effects model followed by the Bonferroni correction was used. The sample size was calculated using an a priori *one‐way* ANOVA power analysis with the software G*Power Version 3.1.4 (Faul et al. 2007) (further details: Data S1).

## 3 Results

### 3.1 Chronic Starvation Leads to AN‐Related Symptoms

First, we investigated whether chronic starvation leads to AN‐related symptoms, such as hyperactivity and amenorrhea, and whether refeeding leads to the reversion of these symptoms (Figure 2).

SIH mice reached a 25% body weight loss on average on day 15 (SIH: 12.17 g ± 0.13; red dotted line, Figure 2C). During acute and chronic starvation, the body weight of SIH mice was significantly reduced compared to the weight of controls. After 4 days of refeeding, the SIH mice reached the same body weight as the control mice. Acute and chronic starvation led to hyperactivity in SIH mice (Figure 2D). In contrast, refeeding led to a significant decrease in running activity, especially during the first 7 days of refeeding. Figure 2E shows the incidence of mice with a fertile phase within a 4‐day block. At the beginning of chronic starvation, none of the SIH mice had a regular estrous cycle, and regular estrous cycles remained absent during chronic starvation, highly suggestive of amenorrhea in all mice. In comparison, during the refeeding phase, regular estrous cycles reappeared. In summary, SIH mice demonstrate the key AN symptoms of weight loss, hyperactivity, and amenorrhea.

### 3.2 Chronic Starvation Leads to Increased Serum Neurofilament Light Chain Levels and Decreased Cerebral Cortex, Hippocampal, and Corpus Callosum Volume

**FIGURE 3:**
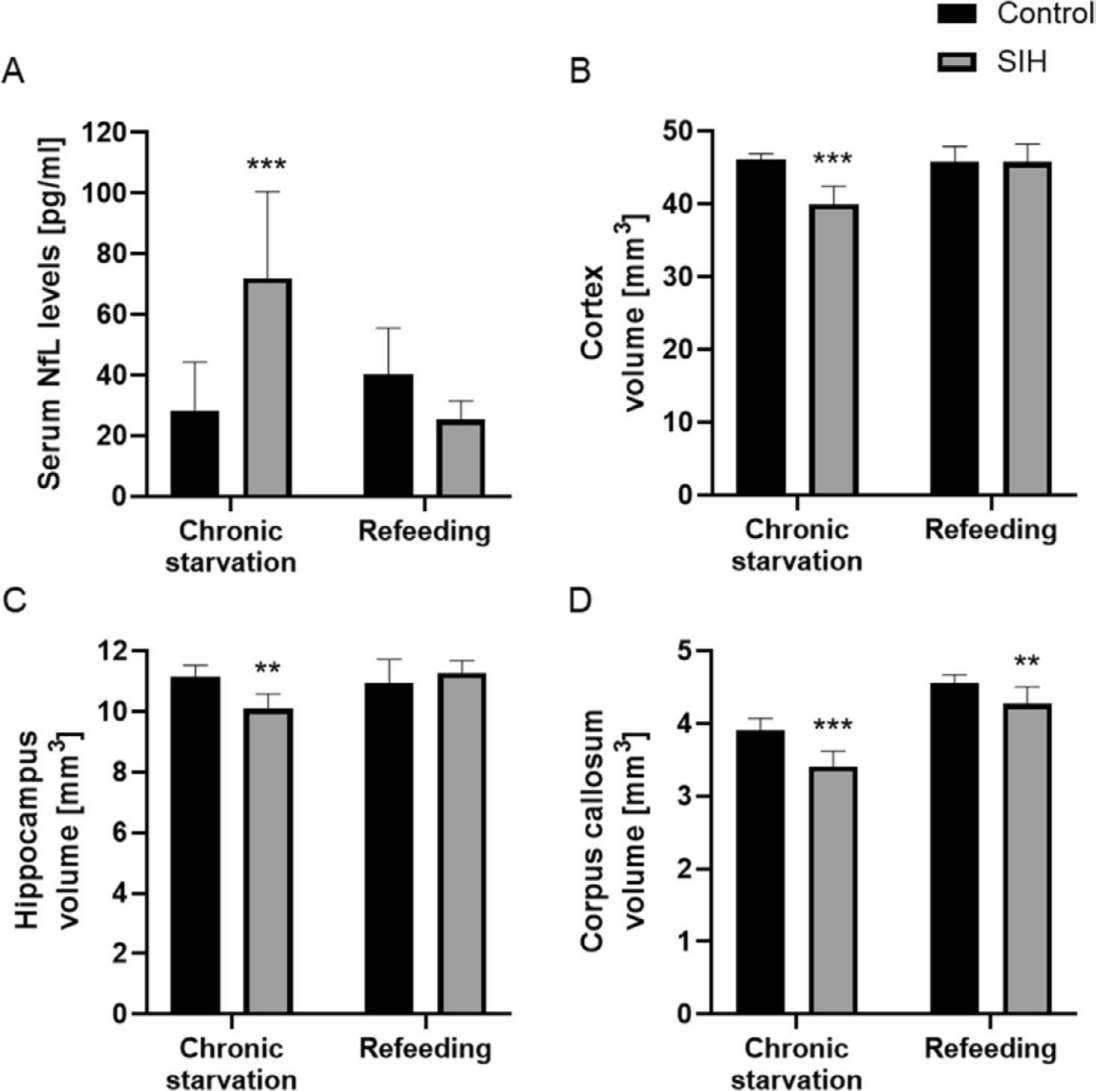
Chronic starvation leads to increased serum NfL levels and decreased cerebral cortex, hippocampal, and corpus callosum volumes. (A) After chronic starvation, serum NfL levels were increased in SIH mice compared to corresponding controls. After refeeding, there was no significant increase in comparison to controls. (B) While the volume of the cerebral cortex was reduced after chronic starvation, no significant reduction was observed in SIH mice after refeeding. (C) After chronic starvation, the hippocampal volume of SIH mice was reduced, which normalized after refeeding. (D) The volume of the corpus callosum in SIH mice was reduced after chronic starvation. This reduction persisted after refeeding. (A–D) All parameters from each mouse were determined at the end of the experiment. Two‐way ANOVA was performed to compare serum NfL levels and volumes of cortex, hippocampus, and corpus callosum between SIH and control mice after chronic starvation and refeeding, **p ≤ 0.01, ***p ≤ 0.001.

Next, we investigated whether chronic starvation leads to changes in brain volume and whether these changes are paralleled by altered serum NfL levels in the SIH model (Figure 3). Moreover, we investigated whether refeeding reverses these effects.

As demonstrated in Figure 3A, after chronic starvation, serum NfL levels in SIH mice were significantly increased in comparison to control mice. At the end of the refeeding period, serum NfL levels in SIH mice normalized and were not altered in comparison to control mice.

To investigate whether brain volume changes differed in relation to different brain regions, the cortex, hippocampus, and corpus callosum were analyzed. While cortex volume decreased in chronically‐starved SIH mice, there was no difference between SIH and control mice after refeeding (Figure 3B). Chronic starvation led to a decrease in hippocampal (Figure 3C) and corpus callosum volumes (Figure 3D). After refeeding, hippocampal volume was normalized in SIH mice compared to controls. Conversely, corpus callosum volume was still decreased in SIH mice compared to controls after refeeding (Figure 3D). To summarize, increased serum NfL levels are paralleled by brain atrophy in SIH mice.

### 3.3 Chronic Starvation Leads to a Decrease in Anxiety‐Like Behavior in the SIH Model

**FIGURE 4:**
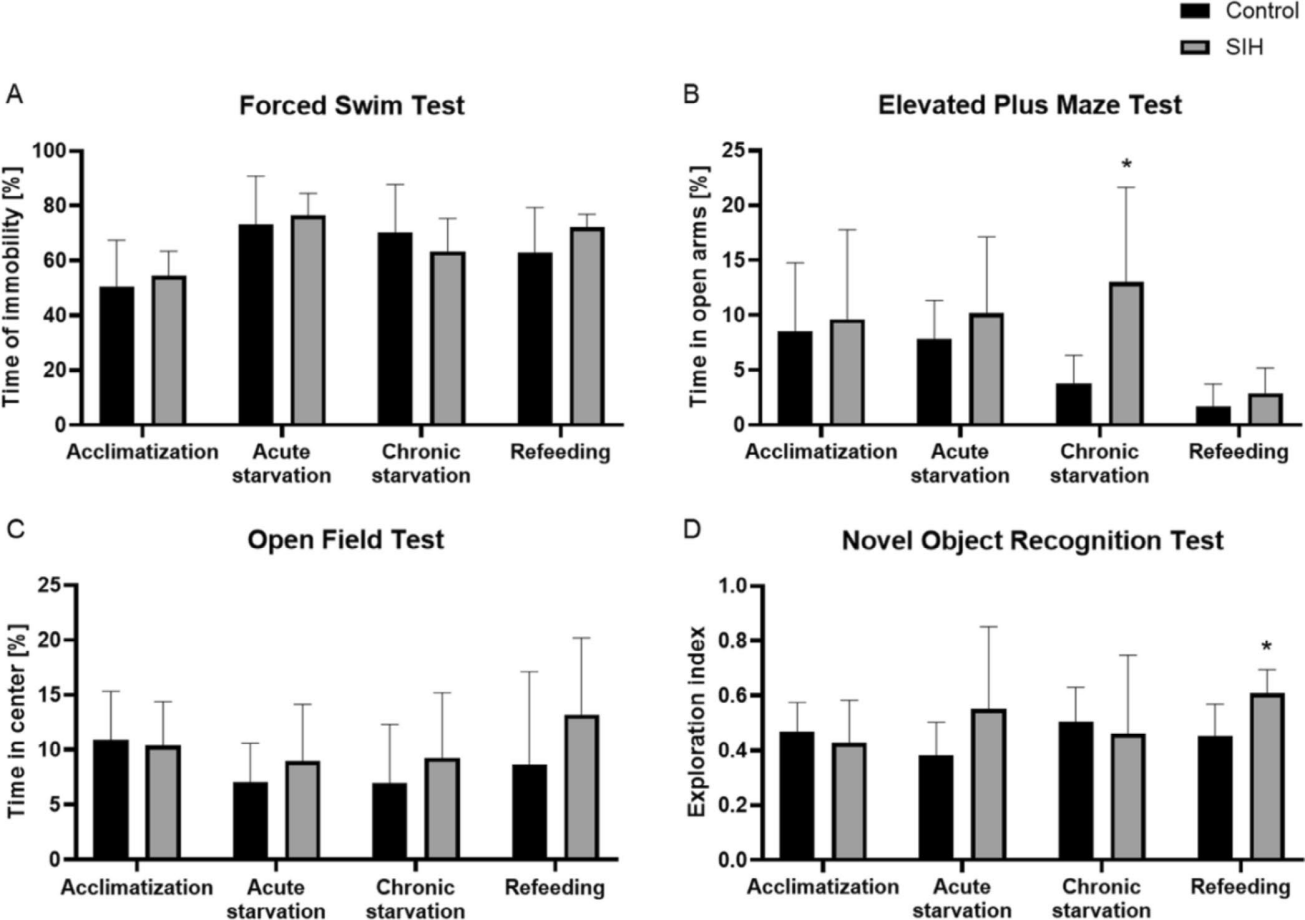
Chronic starvation reduces anxiety‐like behavior without affecting depressive‐like behavior or recognition memory. After refeeding, recognition memory is more pronounced. (A) Time of immobility (in percent) during the forced swim test did not change in SIH compared to control mice. (B) Time spent in the open arms (in percent) in the elevated plus maze test was increased after chronic starvation compared to controls. (C) Time spent in the center (in percent) during the open field test did not change in SIH compared to control mice. (D) The exploration index was defined as the exploration time of a new object divided by the total exploration time of both objects. After refeeding, the exploration index was increased compared to controls. Two‐way ANOVA was performed to compare time of immobility, time in open arms, time in center, and exploration index between SIH and control mice for the different phases (acclimatization, acute starvation, chronic starvation and refeeding), *p ≤ 0.05.

Next, we analyzed whether increased serum NfL levels and brain atrophy are paralleled by changes in behavior (Figure 4). To this end, different behavioral tests were applied to estimate levels of depression (FST), anxiety (EPM test and OF test), and recognition memory (NOR test).

Throughout the experiment, starvation did not affect immobility time in the FST, indicating no change in depressive‐like behavior (Figure 4A). To assess the effects of starvation on anxiety‐like behavior, the EPM test and OF test were performed (Figure 4B,C). The EPM test showed a significant increase in time spent in open arms in mice after chronic starvation, indicating decreased anxiety‐like behavior. After refeeding, there was no difference in time spent in open arms between SIH and control mice. The OF test showed no change in time spent in the center for SIH mice compared to controls throughout the experiment. Recognition memory was evaluated using the NOR test (Figure 4D). There was no significant change in the exploration index in SIH mice compared to controls after starvation. However, after refeeding, the exploration index was significantly higher in SIH mice compared to controls. In summary, chronic starvation decreased anxiety‐like behavior but did not affect depressive‐like behavior or recognition memory. After refeeding, SIH mice showed improved recognition memory.

### 3.4 Chronic Starvation Leads to Decreased Microglia Cell Density and Changes in Morphology in the Cerebral Cortex

**FIGURE 5:**
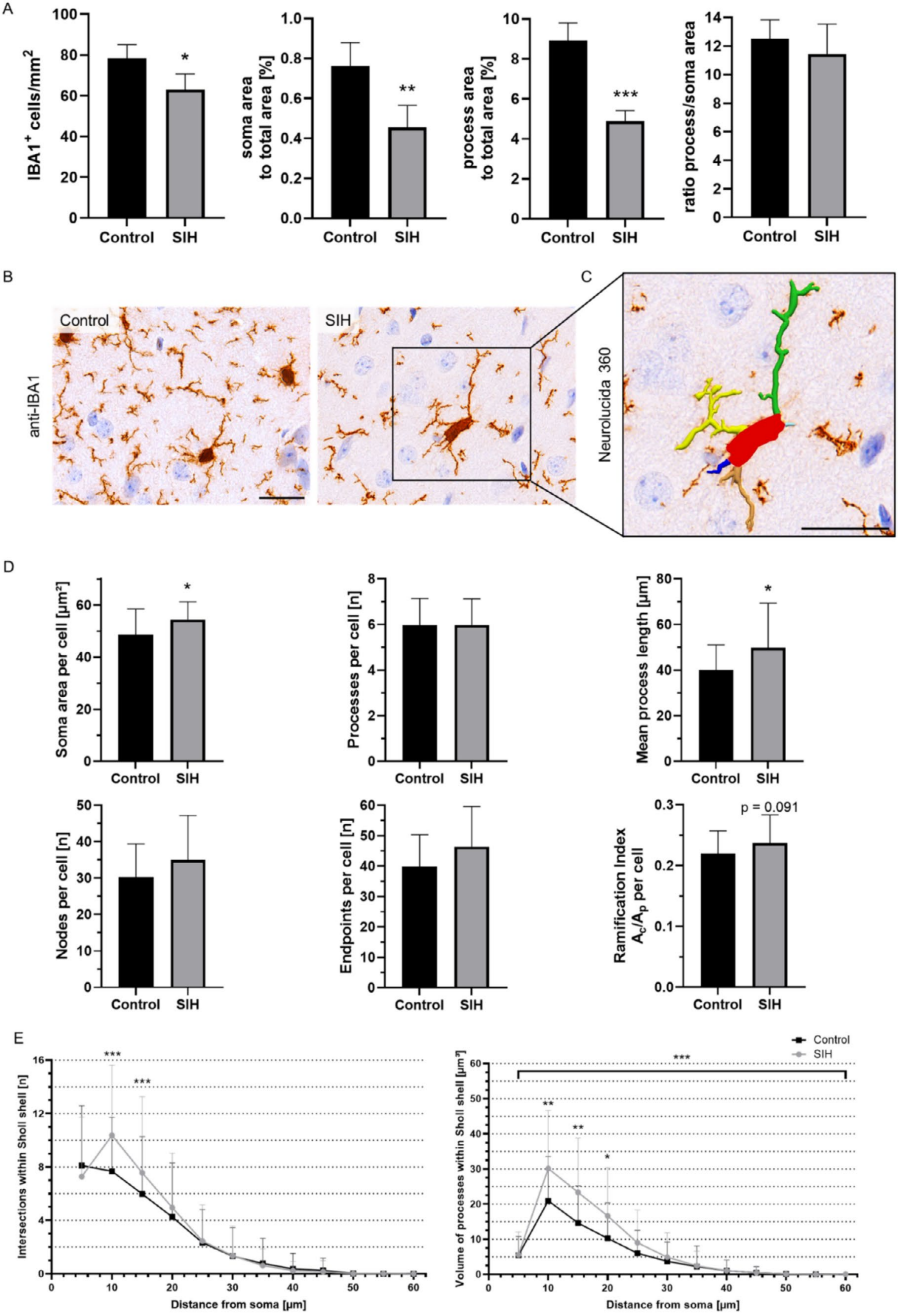
Chronic starvation induces a reduction in IBA1^+^ cell density and changes in microglial morphology. (A) The density of IBA1^+^ cells, the area of somas, and the area of processes to total area in the cerebral cortex of SIH mice were decreased in comparison to control mice after chronic starvation. The ratio of process to soma area did not change after chronic starvation. (B) Representative images of the anti‐IBA1 microglia staining in control and SIH mice. (C) Magnified SIH microglial cell reconstructed with Neurolucida 360 for morphological analysis. (D) After chronic starvation, soma area per cell and mean process length of SIH mice were increased compared to controls. The number of processes, nodes, and endpoints per cell did not change, while a trend to an increased ramification index was observed in SIH mice after chronic starvation. (E) Intersections and volume of processes were changed within different Sholl shells. Scale bars: 25 μm; Two‐sided Student's *t*‐test was performed to compare IBA1^+^ cell density and soma area per cell between SIH and control mice; Mann–Whitney test was performed to compare processes per cell, mean process length, nodes, endpoints, and ramification index per cell between SIH and control mice; mixed‐effects model was performed to compare intersections and process volume within Sholl shell between SIH and control mice and between distances from soma, *p ≤ 0.05, **p ≤ 0.01, ***p ≤ 0.001.

We further investigated whether brain atrophy and elevated serum NfL levels reflect underlying neurodegeneration, focusing on alterations in microglial cells (Figure 5). As neuronal dysfunction is associated with changes in microglial cell density and morphology, IBA1^+^ microglial cells were analyzed as an indirect marker of neuronal damage.

After chronic starvation, the density of IBA1^+^ cells in the cerebral cortex was decreased in SIH mice (Figure 5A,B). In addition, soma area and process area to the total cortex area were decreased in SIH mice. The ratio of process to soma area remained unchanged after chronic starvation in SIH mice compared to controls.

Furthermore, microglial cell morphology measured with Neurolucida 360 (Figure 5C) was altered after chronic starvation, as indicated by an increased soma area per cell and mean process length in SIH mice (Figure 5D). While the number of processes, nodes, and endpoints per cell did not change, a trend toward an increased RI was observed in SIH mice after chronic starvation.

Sholl analysis revealed no significant change in the number of intersections across all Sholl shells. However, analysis of the individual Sholl shells demonstrated that the number of intersections increased at a distance of 10 and 15 μm from the soma in SIH mice compared to controls (Figure 5E).

In addition, the volume of processes across all Sholl shells was increased in the SIH group after chronic starvation, highlighted by an increased volume at 10 μm distance from the soma. In summary, chronic starvation led to decreased IBA1^+^ cell density and morphology changes, as indicated by increased soma area, mean process length, and process volume.

## 4 Discussion

AN, a psychiatric disorder characterized by extreme weight loss and distorted body image (Moskowitz and Weiselberg 2017; Herpertz‐Dahlmann 2015), is associated with extensive locomotor activity (Moskowitz and Weiselberg 2017; Herpertz‐Dahlmann 2015). This study aimed to investigate whether the SIH model is a valid method for analyzing potential neurodegeneration using NfL.

As previously described, SIH mice demonstrated the AN‐related symptoms of hyperactivity and amenorrhea after chronic starvation (Staffeld et al. 2023). Additionally, our recent findings demonstrated that a phase of refeeding can reverse these symptoms. Running activity increased during starvation, initially decreased during the phase of refeeding, and then gradually returned to the same level as the control group. The initial decrease was probably due to an increase in food intake immediately after refeeding, as already described in a previous study (Trinh et al. 2023). The symptom of amenorrhea was observed in all SIH mice with the beginning of chronic starvation, indicating that it could be a secondary dysfunction of the hypothalamus. As no change in glial cell densities was observed in the hypothalamus, neuronal damage seems to be a potential mechanism underlying this phenomenon (Zimmermann et al. 2023). This is consistent with the findings of a study with *anx/anx* mice, which showed degeneration of hypothalamic arcuate neurons (Nilsson et al. 2011). Furthermore, an estrous cycle was not detected immediately at the start of refeeding in the SIH mice, however with a time delay. This could indicate the involvement of the hypothalamic–pituitary–ovarian (HPO) axis, which leads to the corresponding time delay due to the feedback mechanisms involved.

To our knowledge, we demonstrated for the first time in an AN‐mimicking animal model that NfL levels increase after chronic starvation, potentially due to neuronal damage. This finding is consistent with the results of previous studies that observed increased serum levels of NfL in AN patients (Nilsson et al. 2019; Hellerhoff et al. 2021). Furthermore, we showed that reduced cerebral cortex volume was paralleled by increased NfL levels in SIH mice after chronic starvation. This is in line with the findings that elevated NfL levels are associated with reduced cortical thickness in AN patients in several brain regions (Hellerhoff et al. 2023). Another study in AN patients demonstrated that total N‐acetyl aspartate levels are negatively associated with NfL levels, indicating neuronal damage, further supporting previous findings (Doose et al. 2023). A further study has already shown that neurodegeneration is evident in the medial prefrontal cortex in a DIA rat model (Reyes‐Ortega et al. 2020). Furthermore, we showed that volume reduction was not limited to the cerebral cortex, as hippocampal and corpus callosum volumes also decreased, mirroring global volumetric brain changes found in patients with AN (Maier et al. 2022; Connan et al. 2006). After refeeding, normalized NfL levels in SIH mice were paralleled by normalized cerebral cortex and hippocampal volume compared to controls, consistent with our previous study in rats (Frintrop et al. 2019). This conforms to studies with AN patients showing reversible NfL levels after body weight rehabilitation (Hellerhoff et al. 2021; Doose et al. 2022). This is further supported by a study in AN patients which showed that reductions in hippocampal subfield volumes are largely reversible with weight recovery (Bahnsen et al. 2024). Only corpus callosum volume remained reduced after refeeding, which is also consistent with our previous study (Frintrop et al. 2019). This could be due to lasting effects on white matter integrity that slow the reversion of corpus callosum volume. Furthermore, a longer refeeding period may be required to fully restore energy deficits and support myelin synthesis (Asadollahi et al. 2024).

Behavioral tests were used to assess depressive‐like and anxiety‐like behavior, as well as recognition memory. Our findings suggest that chronic starvation does not affect depressive‐like behavior. However, studies have shown that chronic starvation induces both depressogenic and antidepressant effects, leading to both increased and decreased immobility time (Jahng et al. 2007; Wang et al. 2021). We demonstrated that chronic starvation leads to decreased anxiety‐like behavior, indicating an anxiolytic effect of starvation. Previous studies on anxiety‐like behavior in rodent models of chronic starvation are associated with both increases and decreases in anxiety‐like behavior (Jahng et al. 2007; Wang et al. 2021; Schwenzer et al. 2022; Wable et al. 2015; Yamamoto et al. 2009). Schwenzer and colleagues showed a reduction in anxiety‐like behavior measured by the EPM test in SIH rats, which is consistent with our results (Schwenzer et al. 2022). Furthermore, anxiety‐like behavior observed exclusively in the EPM test may be linked to a foraging drive that encourages the exploration of novel spaces (Hebebrand et al. 2022). We demonstrated in a previous study that SIH rats had impaired memory in a NOR task (Paulukat et al. 2016). Another study showed that chronically‐starved rats after refeeding still had impaired memory, suggesting a long‐lasting effect of starvation (Boersma et al. 2016). In contrast, our study did not show any effect in this test after starvation. However, improved recognition memory was observed in SIH mice after refeeding, suggesting that starvation might have lasting effects.

In addition, in neurodegenerative diseases, neuronal damage is associated with glial cell changes, particularly microglia, the brain's resident immune cells. Neuronal damage can affect microglia density and phenotype in a complex process called microgliosis (Zrzavy et al. 2017). Accordingly, this study investigated the role of microglia, demonstrating that chronic starvation led to a reduced density of IBA1^+^ cells. This is further supported by the finding of reduced areas of processes and soma relative to total cortex areas after chronic starvation in SIH mice. This was consistent with our previous study in SIH mice, which showed a reduction in IBA1^+^ cells in the corpus callosum (Zimmermann et al. 2023). Furthermore, a study indicating reduced neuroinflammation in ABA rats is also consistent with our finding of reduced IBA1^+^ cell density, as this could also be a sign of reduced neuroinflammation (Spero et al. 2024). In contrast, studies in DIA rats revealed increased IBA1^+^ cell numbers in the prefrontal cortex and hippocampus (Reyes‐Ortega et al. 2020; Ragu‐Varman et al. 2019). Therefore, the reaction of microglial cells may differ depending on the type of starvation, duration of starvation, and the specific region of the brain. Additionally, microglial cells directly interact with the synapses of active neurons (Wake et al. 2009) and are involved in the regulation of synaptic plasticity through alterations in synaptic connections (Akiyoshi et al. 2018; Fu et al. 2014). Therefore, microglial dysfunction due to decreased cell density may lead to behavioral changes as indicated by increased anxiety‐like behavior as discussed above.

To assess the extent of microglial activation, microglial cell morphology should be analyzed, as distinct morphological characteristics can differentiate phenotypes associated with specific functional states. While a hyper‐ramified morphology is characterized by increased process length, number, and complexity, an activated microglial cell is characterized by larger somas and thicker, less branched processes (Morrison and Filosa 2013). Furthermore, it is important to consider that intermediate forms between amoeboid and ramified microglia also exist, which may have distinct functions. In addition, studies in animal models of obesity have shown that hypothalamic microglia have an activated morphology (Thaler et al. 2012), indicating that microglia are involved in inflammatory signaling pathways triggered by excessive food intake.

In our study, after chronic starvation increased soma area and process lengths were observed, demonstrating a morphological response of microglial cells to starvation‐induced microenvironment changes. A trend towards an increased RI, more intersections within a 10 μm distance from the soma, and a higher process volume after chronic starvation suggest a shift in microglial phenotype, potentially reflecting a more activated state in response to starvation‐induced stress. However, after chronic starvation, we observed an increased process length and no changes in the number of processes, nodes, and endpoints, indicating that starvation induced microglia to display a specific intermediate morphological phenotype between hyper‐ramified and reactive (Paolicelli et al. 2022). In contrast, Reyes‐Ortega et al. observed morphological changes in microglia as de‐ramified cells in the prefrontal cortex in the DIA model (Reyes‐Ortega et al. 2020). However, a different method was used to assess morphology and, as discussed above, the response of microglial cells may vary depending on the type of starvation, the duration of starvation, and the specific region of the brain. As neuronal dysfunction is associated with morphological changes in microglia, changes after chronic starvation might be an indirect marker of neurodegeneration. Future studies using conditional gene deletion models for targeted manipulation or depletion of microglia may determine their role in the SIH model and their impact on AN‐related symptoms.

In summary, chronic starvation led to AN‐related symptoms of hyperactivity and amenorrhea. Furthermore, increased serum NfL levels after chronic starvation in SIH mice were paralleled by brain atrophy after chronic starvation. In addition, chronic starvation led to a decrease in anxiety‐like behavior in SIH mice, but no changes were observed in depressive‐like behavior or recognition memory. Furthermore, chronic starvation led to decreased microglial cell density and morphological changes. These behavioral and cellular changes may result from a combination of neuronal and glial dysfunction, potentially contributed by blood–brain barrier dysfunction. However, given the reversibility of these findings, early and comprehensive interventions could prevent long‐term neurobiological damage. These interventions should include both rapid weight restoration and psychotherapy to support a more effective recovery process. Nevertheless, as neuronal dysfunction may be involved in the mechanisms underlying the pathophysiology of AN, the SIH model allows us to further investigate its influence on neurons and identify new therapeutic options.

## Author Contributions

**Annelie Zimmermann:** conceptualization, data curation, formal analysis, investigation, methodology, validation, visualization, writing – original draft, writing – review and editing. **Hanna Rupprecht:** data curation, formal analysis, investigation, methodology, visualization. **Stephan Lang:** data curation, formal analysis, investigation, methodology, visualization. **Rickmer Wienecke:** data curation, formal analysis, investigation, methodology, visualization. **Hanna‐Sophia Henschke:** data curation, formal analysis, investigation, methodology, visualization. **Kaja Dickert:** data curation, formal analysis, investigation, methodology, visualization. **Katharina Schuster:** investigation. **Anna Staffeld:** investigation. **Christoph Berger:** validation. **Alexander Dück:** validation. **Michael Kölch:** resources, validation. **Antje Vogelgesang:** formal analysis, methodology. **Matthias Grothe:** formal analysis, methodology. **Leo Heinig:** formal analysis, methodology. **Lukas Wenzel:** formal analysis, methodology. **Markus Kipp:** methodology, resources, writing – review and editing. **Linda Frintrop:** conceptualization, data curation, formal analysis, funding acquisition, investigation, methodology, project administration, resources, supervision, validation, visualization, writing – original draft, writing – review and editing.

## Ethics Statement

All animal procedures were conducted at the Institute of Anatomy at the Rostock University Medical Center, in accordance with EU Directive 2010/63 on the protection of animals used for scientific purposes and the recommendations of the Federation of European Laboratory Animal Science Associations (FELASA). The animal studies were approved by the Review Boards for the Care of Animal Subjects of the district government of Mecklenburg‐Western Pomerania (reference number 7221.3‐1‐005/21). The experiments were reported according to the ARRIVE (Animal Research: Reporting of In Vivo Experiments) guidelines (Du Percie Sert et al. 2020).

## Conflicts of Interest

The authors declare no conflicts of interest.

## Supporting information


**Data S1.** Supporting Information.

Video S1.

## Data Availability

Data may be made available upon sending requests to the corresponding author.

## References

[eat24423-bib-0001] Akiyoshi, R. , H. Wake , D. Kato , et al. 2018. “Microglia Enhance Synapse Activity to Promote Local Network Synchronization.” eNeuro 5: ENEURO.0088. 10.1523/ENEURO.0088-18.2018. 30406198 PMC6220592

[eat24423-bib-0002] Asadollahi, E. , A. Trevisiol , A. S. Saab , et al. 2024. “Oligodendroglial Fatty Acid Metabolism as a Central Nervous System Energy Reserve.” Nature Neuroscience 27: 1934–1944. 10.1038/s41593-024-01749-6. 39251890 PMC11452346

[eat24423-bib-0003] Bacioglu, M. , L. F. Maia , O. Preische , et al. 2016. “Neurofilament Light Chain in Blood and CSF as Marker of Disease Progression in Mouse Models and in Neurodegenerative Diseases.” Neuron 91: 56–66. 10.1016/j.neuron.2016.05.018. 27292537

[eat24423-bib-0004] Bahnsen, K. , M.‐L. Wronski , J. L. Keeler , et al. 2024. “Differential Longitudinal Changes of Hippocampal Subfields in Patients With Anorexia Nervosa.” Psychiatry and Clinical Neurosciences 78: 186–196. 10.1111/pcn.13626. 38018338 PMC11488614

[eat24423-bib-0005] Beecken, M. , L. Baumann , E. Vankriekelsvenne , et al. 2023. “The Cuprizone Mouse Model: A Comparative Study of Cuprizone Formulations From Different Manufacturers.” International Journal of Molecular Sciences 24: 10564. 10.3390/ijms241310564. 37445742 PMC10341492

[eat24423-bib-0006] Boersma, G. J. , Y. Treesukosol , Z. A. Cordner , et al. 2016. “Exposure to Activity‐Based Anorexia Impairs Contextual Learning in Weight‐Restored Rats Without Affecting Spatial Learning, Taste, Anxiety, or Dietary‐Fat Preference.” International Journal of Eating Disorders 49, no. 2: 167–179. 10.1002/eat.22489. 26711541 PMC4777973

[eat24423-bib-0007] Castro‐Fornieles, J. , X. Caldú , S. Andrés‐Perpiñá , et al. 2010. “A Cross‐Sectional and Follow‐Up Functional MRI Study With a Working Memory Task in Adolescent Anorexia Nervosa.” Neuropsychologia 48: 4111–4116. 10.1016/j.neuropsychologia.2010.10.003. 20933530

[eat24423-bib-0008] Connan, F. , F. Murphy , S. E. J. Connor , et al. 2006. “Hippocampal Volume and Cognitive Function in Anorexia Nervosa.” Psychiatry Research 146: 117–125. 10.1016/j.pscychresns.2005.10.006. 16510268

[eat24423-bib-0009] Doose, A. , I. Hellerhoff , F. I. Tam , et al. 2022. “Neural and Glial Damage Markers in Women After Long‐Term Weight‐Recovery From Anorexia Nervosa.” Psychoneuroendocrinology 135: 105576. 10.1016/j.psyneuen.2021.105576. 34781223

[eat24423-bib-0010] Doose, A. , F. I. Tam , I. Hellerhoff , et al. 2023. “Triangulating Brain Alterations in Anorexia Nervosa: A Multimodal Investigation of Magnetic Resonance Spectroscopy, Morphometry and Blood‐Based Biomarkers.” Translational Psychiatry 13: 277. 10.1038/s41398-023-02580-6. 37573444 PMC10423271

[eat24423-bib-0011] Du Percie Sert, N. , A. Ahluwalia , S. Alam , et al. 2020. “Reporting Animal Research: Explanation and Elaboration for the ARRIVE Guidelines 2.0.” PLoS Biology 18, no. 7: e3000411. 10.1371/journal.pbio.3000411. 32663221 PMC7360025

[eat24423-bib-0012] Faul, F. , E. Erdfelder , A.‐G. Lang , and A. Buchner . 2007. “G*Power 3: A Flexible Statistical Power Analysis Program for the Social, Behavioral, and Biomedical Sciences.” Behavior Research Methods 39: 175–191. 10.3758/bf03193146. 17695343

[eat24423-bib-0013] Franklin, K. B. J. , and G. Paxinos . 2005. The Mouse Brain in Stereotaxic Coordinates. 2nd ed. Acad. Press.

[eat24423-bib-0014] Frintrop, L. , S. Trinh , J. Liesbrock , et al. 2019. “The Reduction of Astrocytes and Brain Volume Loss in Anorexia Nervosa‐The Impact of Starvation and Refeeding in a Rodent Model.” Translational Psychiatry 9: 159. 10.1038/s41398-019-0493-7. 31164627 PMC6548775

[eat24423-bib-0015] Frintrop, L. , S. Trinh , J. Liesbrock , et al. 2018. “Establishment of a Chronic Activity‐Based Anorexia Rat Model.” Journal of Neuroscience Methods 293: 191–198. 10.1016/j.jneumeth.2017.09.018. 28970163

[eat24423-bib-0016] Fu, R. , Q. Shen , P. Xu , J. J. Luo , and Y. Tang . 2014. “Phagocytosis of Microglia in the Central Nervous System Diseases.” Molecular Neurobiology 49: 1422–1434. 10.1007/s12035-013-8620-6. 24395130 PMC4012154

[eat24423-bib-0017] Fuglset, T. S. , N. I. Landrø , D. L. Reas , and Ø. Rø . 2016. “Functional Brain Alterations in Anorexia Nervosa: A Scoping Review.” Journal of Eating Disorders 4: 32. 10.1186/s40337-016-0118-y. 27933159 PMC5125031

[eat24423-bib-0018] Gabloffsky, T. , S. Gill , A. Staffeld , et al. 2022. “Food Restriction in Mice Induces Food‐Anticipatory Activity and Circadian‐Rhythm‐Related Activity Changes.” Nutrients 14: 5252. 10.3390/nu14245252. 36558413 PMC9782400

[eat24423-bib-0019] Gaetani, L. , K. Blennow , P. Calabresi , M. Di Filippo , L. Parnetti , and H. Zetterberg . 2019. “Neurofilament Light Chain as a Biomarker in Neurological Disorders.” Journal of Neurology, Neurosurgery, and Psychiatry 90: 870–881. 10.1136/jnnp-2018-320106. 30967444

[eat24423-bib-0020] Galmiche, M. , P. Déchelotte , G. Lambert , and M. P. Tavolacci . 2019. “Prevalence of Eating Disorders Over the 2000‐2018 Period: A Systematic Literature Review.” American Journal of Clinical Nutrition 109: 1402–1413. 10.1093/ajcn/nqy342. 31051507

[eat24423-bib-0021] Hall, C. S. 1934. “Emotional Behavior in the Rat. I. Defecation and Urination as Measures of Individual Differences in Emotionality.” Journal of Comparative Psychology 18: 385–403. 10.1037/h0071444.

[eat24423-bib-0022] Hebebrand, J. , T. Hildebrandt , H. Schlögl , et al. 2022. “The Role of Hypoleptinemia in the Psychological and Behavioral Adaptation to Starvation: Implications for Anorexia Nervosa.” Neuroscience and Biobehavioral Reviews 141: 104807. 10.1016/j.neubiorev.2022.104807. 35931221

[eat24423-bib-0023] Hellerhoff, I. , F. Bernardoni , K. Bahnsen , et al. 2023. “Serum Neurofilament Light Concentrations Are Associated With Cortical Thinning in Anorexia Nervosa.” Psychological Medicine 53: 7053–7061. 10.1017/S0033291723000387. 36967674 PMC10719626

[eat24423-bib-0024] Hellerhoff, I. , J. A. King , F. I. Tam , et al. 2021. “Differential Longitudinal Changes of Neuronal and Glial Damage Markers in Anorexia Nervosa After Partial Weight Restoration.” Translational Psychiatry 11: 86. 10.1038/s41398-021-01209-w. 33558486 PMC7870648

[eat24423-bib-0025] Heppner, F. L. , K. Roth , R. Nitsch , and N. P. Hailer . 1998. “Vitamin E Induces Ramification and Downregulation of Adhesion Molecules in Cultured Microglial Cells.” Glia 22: 180–188. 10.1002/(SICI)1098-1136(199802)22:2<180:AID-GLIA9>3.0.CO;2-B. 9537838

[eat24423-bib-0026] Herpertz‐Dahlmann, B. 2015. “Adolescent Eating Disorders: Update on Definitions, Symptomatology, Epidemiology, and Comorbidity.” Child and Adolescent Psychiatric Clinics of North America 24: 177–196. 10.1016/j.chc.2014.08.003. 25455581

[eat24423-bib-0027] Jagielska, G. , and I. Kacperska . 2017. “Outcome, Comorbidity and Prognosis in Anorexia Nervosa.” Psychiatria Polska 51: 205–218. 10.12740/PP/64580. 28581532

[eat24423-bib-0028] Jahng, J. W. , J. G. Kim , H. J. Kim , B.‐T. Kim , D.‐W. Kang , and J.‐H. Lee . 2007. “Chronic Food Restriction in Young Rats Results in Depression‐ and Anxiety‐Like Behaviors With Decreased Expression of Serotonin Reuptake Transporter.” Brain Research 1150: 100–107. 10.1016/j.brainres.2007.02.080. 17383614

[eat24423-bib-0029] Kawakami, I. , S. Iritani , Y. Riku , et al. 2022. “Neuropathological Investigation of Patients With Prolonged Anorexia Nervosa.” Psychiatry and Clinical Neurosciences 76: 187–194. 10.1111/pcn.13340. 35167165 PMC9314851

[eat24423-bib-0030] Keski‐Rahkonen, A. , and L. Mustelin . 2016. “Epidemiology of Eating Disorders in Europe: Prevalence, Incidence, Comorbidity, Course, Consequences, and Risk Factors.” Current Opinion in Psychiatry 29: 340–345. 10.1097/YCO.0000000000000278. 27662598

[eat24423-bib-0031] Khalil, M. , C. E. Teunissen , M. Otto , et al. 2018. “Neurofilaments as Biomarkers in Neurological Disorders.” Nature Reviews. Neurology 14: 577–589. 10.1038/s41582-018-0058-z. 30171200

[eat24423-bib-0032] Kogel, V. , S. Trinh , N. Gasterich , C. Beyer , and J. Seitz . 2021. “Long‐Term Glucose Starvation Induces Inflammatory Responses and Phenotype Switch in Primary Cortical Rat Astrocytes.” Journal of Molecular Neuroscience 71: 2368–2382. 10.1007/s12031-021-01800-2. 33580474 PMC8585803

[eat24423-bib-0033] Loeffler, T. , I. Schilcher , S. Flunkert , and B. Hutter‐Paier . 2020. “Neurofilament‐Light Chain as Biomarker of Neurodegenerative and Rare Diseases With High Translational Value.” Frontiers in Neuroscience 14: 579. 10.3389/fnins.2020.00579. 32595447 PMC7300175

[eat24423-bib-0034] Lueptow, L. M. 2017. “Novel Object Recognition Test for the Investigation of Learning and Memory in Mice.” Journal of Visualized Experiments 30: 55718. 10.3791/55718. PMC5614391 28892027

[eat24423-bib-0035] Luine, V. N. , and M. Frankfurt . 2012. “Estrogens Facilitate Memory Processing Through Membrane Mediated Mechanisms and Alterations in Spine Density.” Frontiers in Neuroendocrinology 33: 388–402. 10.1016/j.yfrne.2012.07.004. 22981654 PMC3496031

[eat24423-bib-0036] Maier, S. , A. Joos , L. van Tebartz Elst , et al. 2022. “Reduced Structural Connectivity in the Corpus Callosum in Patients With Anorexia Nervosa.” European Eating Disorders Review 30, no. 4: 341–352. 10.1002/erv.2894. 35306728

[eat24423-bib-0037] Maltais, L. J. , P. W. Lane , and W. G. Beamer . 1984. “Anorexia, a Recessive Mutation Causing Starvation in Preweanling Mice.” Journal of Heredity 75: 468–472. 10.1093/oxfordjournals.jhered.a109987. 6595305

[eat24423-bib-0038] Martin, F. 1958. “Pathology of Neurological & Psychiatric Aspects of Various Deficiency Manifestations With Digestive & Neuro‐Endocrine Disorders: Study of the Changes of the Central Nervous System in 2 Cases of Anorexia in Young Girls (So‐Called Mental Anorexia).” Acta Neurologica et Psychiatrica Belgica 58: 816–830. 13605672

[eat24423-bib-0039] McCormick, L. M. , P. K. Keel , M. C. Brumm , et al. 2008. “Implications of Starvation‐Induced Change in Right Dorsal Anterior Cingulate Volume in Anorexia Nervosa.” International Journal of Eating Disorders 41, no. 7: 602–610. 10.1002/eat.20549. 18473337 PMC3652574

[eat24423-bib-0040] Montgomery, K. C. 1955. “The Relation Between Fear Induced by Novel Stimulation and Exploratory Behavior.” Journal of Comparative and Physiological Psychology 48: 254–260. 10.1037/h0043788. 13252152

[eat24423-bib-0041] Morrison, H. W. , and J. A. Filosa . 2013. “A Quantitative Spatiotemporal Analysis of Microglia Morphology During Ischemic Stroke and Reperfusion.” Journal of Neuroinflammation 10: 4. 10.1186/1742-2094-10-4. 23311642 PMC3570327

[eat24423-bib-0042] Moskowitz, L. , and E. Weiselberg . 2017. “Anorexia Nervosa/Atypical Anorexia Nervosa.” Current Problems in Pediatric and Adolescent Health Care 47: 70–84. 10.1016/j.cppeds.2017.02.003. 28532965

[eat24423-bib-0043] Neumärker, K. J. , U. Dudeck , U. Meyer , U. Neumärker , E. Schulz , and B. Schönheit . 1997. “Anorexia Nervosa and Sudden Death in Childhood: Clinical Data and Results Obtained From Quantitative Neurohistological Investigations of Cortical Neurons.” European Archives of Psychiatry and Clinical Neuroscience 247: 16–22. 10.1007/BF02916248. 9088801

[eat24423-bib-0044] Nilsson, I. A. K. , V. Millischer , V. D. Karrenbauer , et al. 2019. “Plasma Neurofilament Light Chain Concentration Is Increased in Anorexia Nervosa.” Translational Psychiatry 9, no. 1: 180. 10.1038/s41398-019-0518-2. 31371701 PMC6675786

[eat24423-bib-0045] Nilsson, I. A. K. , S. Thams , C. Lindfors , et al. 2011. “Evidence of Hypothalamic Degeneration in the Anorectic Anx/Anx Mouse.” Glia 59: 45–57. 10.1002/glia.21075. 20967882

[eat24423-bib-0046] Paolicelli, R. C. , A. Sierra , B. Stevens , et al. 2022. “Microglia States and Nomenclature: A Field at Its Crossroads.” Neuron 110: 3458–3483. 10.1016/j.neuron.2022.10.020. 36327895 PMC9999291

[eat24423-bib-0047] Paulukat, L. , L. Frintrop , J. Liesbrock , et al. 2016. “Memory Impairment Is Associated With the Loss of Regular Oestrous Cycle and Plasma Oestradiol Levels in an Activity‐Based Anorexia Animal Model.” World Journal of Biological Psychiatry 17: 274–284. 10.3109/15622975.2016.1173725. 27160428

[eat24423-bib-0048] Porsolt, R. D. , G. Anton , N. Blavet , and M. Jalfre . 1978. “Behavioural Despair in Rats: A New Model Sensitive to Antidepressant Treatments.” European Journal of Pharmacology 47: 379–391. 10.1016/0014-2999(78)90118-8. 204499

[eat24423-bib-0049] Porsolt, R. D. , A. Bertin , and M. Jalfre . 1977. “Behavioral Despair in Mice: A Primary Screening Test for Antidepressants.” Archives Internationales de Pharmacodynamie et de Thérapie 229: 327–336. 596982

[eat24423-bib-0050] Ragu‐Varman, D. , M. Macedo‐Mendoza , F. E. Labrada‐Moncada , et al. 2019. “Anorexia Increases Microglial Density and Cytokine Expression in the Hippocampus of Young Female Rats.” Behavioural Brain Research 363: 118–125. 10.1016/j.bbr.2019.01.042. 30690107

[eat24423-bib-0051] Reddaway, J. , P. E. Richardson , R. J. Bevan , J. Stoneman , and M. Palombo . 2023. “Microglial Morphometric Analysis: So Many Options, So Little Consistency.” Frontiers in Neuroinformatics 17: 1211188. 10.3389/fninf.2023.1211188. 37637472 PMC10448193

[eat24423-bib-0052] Reyes‐Haro, D. , F. E. Labrada‐Moncada , D. R. Varman , et al. 2016. “Anorexia Reduces GFAP+ Cell Density in the Rat Hippocampus.” Neural Plasticity 2016: 2426413. 10.1155/2016/2426413. 27579183 PMC4992534

[eat24423-bib-0053] Reyes‐Ortega, P. , D. Ragu Varman , V. M. Rodríguez , and D. Reyes‐Haro . 2020. “Anorexia Induces a Microglial Associated Pro‐Inflammatory Environment and Correlates With Neurodegeneration in the Prefrontal Cortex of Young Female Rats.” Behavioural Brain Research 392: 112606. 10.1016/j.bbr.2020.112606. 32387351

[eat24423-bib-0054] Routtenberg, A. , and A. W. Kuznesof . 1967. “Self‐Starvation of Rats Living in Activity Wheels on a Restricted Feeding Schedule.” Journal of Comparative and Physiological Psychology 64: 414–421. 10.1037/h0025205. 6082873

[eat24423-bib-0055] Salbach‐Andrae, H. , K. Lenz , N. Simmendinger , N. Klinkowski , U. Lehmkuhl , and E. Pfeiffer . 2008. “Psychiatric Comorbidities Among Female Adolescents With Anorexia Nervosa.” Child Psychiatry and Human Development 39: 261–272. 10.1007/s10578-007-0086-1. 17987378

[eat24423-bib-0056] Schneider, C. A. , W. S. Rasband , and K. W. Eliceiri . 2012. “NIH Image to ImageJ: 25 Years of Image Analysis.” Nature Methods 9: 671–675. 10.1038/nmeth.2089. 22930834 PMC5554542

[eat24423-bib-0057] Schwenzer, C. , C. Voelz , V. Kogel , et al. 2022. “Fear and Food: Anxiety‐Like Behavior and the Susceptibility to Weight Loss in an Activity‐Based Anorexia Rat Model.” Clinical and Translational Science 15: 889–898. 10.1111/cts.13196. 34793620 PMC9010269

[eat24423-bib-0058] Seitz, J. , K. Bühren , G. G. von Polier , N. Heussen , B. Herpertz‐Dahlmann , and K. Konrad . 2014. “Morphological Changes in the Brain of Acutely Ill and Weight‐Recovered Patients With Anorexia Nervosa. A Meta‐Analysis and Qualitative Review.” Zeitschrift für Kinder‐ und Jugendpsychiatrie und Psychotherapie 42: 7–17. 10.1024/1422-4917/a000265. 24365959

[eat24423-bib-0059] Silén, Y. , and A. Keski‐Rahkonen . 2022. “Worldwide Prevalence of DSM‐5 Eating Disorders Among Young People.” Current Opinion in Psychiatry 35: 362–371. 10.1097/YCO.0000000000000818. 36125216

[eat24423-bib-0060] Spero, V. , M. Scherma , S. D'Amelio , et al. 2024. “Activity‐Based Anorexia (ABA) Model: Effects on Brain Neuroinflammation, Redox Balance and Neuroplasticity During the Acute Phase.” Neurochemistry International 180: 105842. 10.1016/j.neuint.2024.105842. 39244038

[eat24423-bib-0061] Staffeld, A. , S. Gill , A. Zimmermann , et al. 2023. “Establishment of a Murine Chronic Anorexia Nervosa Model.” Cells 12: 1710. 10.3390/cells12131710. 37443744 PMC10340390

[eat24423-bib-0062] Thaler, J. P. , C.‐X. Yi , E. A. Schur , et al. 2012. “Obesity Is Associated With Hypothalamic Injury in Rodents and Humans.” Journal of Clinical Investigation 122: 153–162. 10.1172/JCI59660. 22201683 PMC3248304

[eat24423-bib-0063] Tose, K. , T. Takamura , M. Isobe , et al. 2024. “Systematic Reduction of Gray Matter Volume in Anorexia Nervosa, but Relative Enlargement With Clinical Symptoms in the Prefrontal and Posterior Insular Cortices: A Multicenter Neuroimaging Study.” Molecular Psychiatry 29: 891–901. 10.1038/s41380-023-02378-4. 38246936 PMC11176065

[eat24423-bib-0064] Trinh, S. , V. Kogel , L. Kneisel , et al. 2023. “Gut Microbiota and Brain Alterations After Refeeding in a Translational Anorexia Nervosa Rat Model.” International Journal of Molecular Sciences 24: 9496. 10.3390/ijms24119496. 37298445 PMC10253567

[eat24423-bib-0065] van Eeden, A. E. , D. van Hoeken , and H. W. Hoek . 2021. “Incidence, Prevalence and Mortality of Anorexia Nervosa and Bulimia Nervosa.” Current Opinion in Psychiatry 34: 515–524. 10.1097/YCO.0000000000000739. 34419970 PMC8500372

[eat24423-bib-0066] Wable, G. S. , J.‐Y. Min , Y.‐W. Chen , and C. Aoki . 2015. “Anxiety Is Correlated With Running in Adolescent Female Mice Undergoing Activity‐Based Anorexia.” Behavioral Neuroscience 129: 170–182. 10.1037/bne0000040. 25730124 PMC4398663

[eat24423-bib-0067] Wake, H. , A. J. Moorhouse , S. Jinno , S. Kohsaka , and J. Nabekura . 2009. “Resting Microglia Directly Monitor the Functional State of Synapses In Vivo and Determine the Fate of Ischemic Terminals.” Journal of Neuroscience 29: 3974–3980. 10.1523/JNEUROSCI.4363-08.2009. 19339593 PMC6665392

[eat24423-bib-0068] Walton, E. , F. Bernardoni , V.‐L. Batury , et al. 2022. “Brain Structure in Acutely Underweight and Partially Weight‐Restored Individuals With Anorexia Nervosa: A Coordinated Analysis by the ENIGMA Eating Disorders Working Group.” Biological Psychiatry 92: 730–738. 10.1016/j.biopsych.2022.04.022. 36031441 PMC12145862

[eat24423-bib-0069] Wang, Q. , Y. Kong , S. Lin , et al. 2021. “The ATP Level in the mPFC Mediates the Antidepressant Effect of Calorie Restriction.” Neuroscience Bulletin 37: 1303–1313. 10.1007/s12264-021-00726-4. 34089507 PMC8423953

[eat24423-bib-0070] Watts, A. G. , and C. N. Boyle . 2010. “The Functional Architecture of Dehydration‐Anorexia.” Physiology and Behavior 100: 472–477. 10.1016/j.physbeh.2010.04.010. 20399797 PMC2886163

[eat24423-bib-0071] Wittekindt, M. , H. Kaddatz , S. Joost , et al. 2022. “Different Methods for Evaluating Microglial Activation Using Anti‐Ionized Calcium‐Binding Adaptor Protein‐1 Immunohistochemistry in the Cuprizone Model.” Cells 11: 1723. 10.3390/cells11111723. 35681418 PMC9179561

[eat24423-bib-0072] Yamamoto, Y. , T. Tanahashi , T. Kawai , et al. 2009. “Changes in Behavior and Gene Expression Induced by Caloric Restriction in C57BL/6 Mice.” Physiological Genomics 39: 227–235. 10.1152/physiolgenomics.00082.2009. 19737990

[eat24423-bib-0073] Zimmermann, A. , N. Böge , K. Schuster , et al. 2023. “Glial Cell Changes in the Corpus Callosum in Chronically‐Starved Mice.” Journal of Eating Disorders 11: 227. 10.1186/s40337-023-00948-z. 38111061 PMC10726510

[eat24423-bib-0074] Zrzavy, T. , S. Hametner , I. Wimmer , O. Butovsky , H. L. Weiner , and H. Lassmann . 2017. “Loss of Homeostatic Microglia and Patterns of Their Activation in Active Multiple Sclerosis.” Brain 140: 1900–1913. 10.1093/brain/awx113. 28541408 PMC6057548

