# Supplementary material for: Increased Serum Neurofilament Light Chain Concentration Associated With Microglial Morphology Changes in Chronically‐Starved Mice
Source: Int J Eat Disord. 2025 Mar 22;58(6):1130–43. doi: 10.1002/eat.24423 (PMC12138848; doi:10.1002/eat.24423)
Supplement: Supplementary file 1 — Data S1. Supporting Information. [file EAT-58-1130-s001.pdf]

2.2 Study Design

As the animals arrive at the age of 4 weeks, which corresponds to 28 days postnatal (P28), the phases can be assigned to the following postnatal days: The end of the acclimatization phase corresponds to P38 (day 10). The end of the acute starvation phase corresponds to P44 (day 16). The end of the chronic starvation phase corresponds to P57 (day 29) and the end of the refeeding phase corresponds to P77 (day 49).

To analyze running wheel activity (previously described in [1,2]) running wheels (11.5 cm in diameter) attached to the top of the cages were used. The running distance per individual mouse was recorded by software (VitalView Activity 1.4, STARR Life Science Corp.) and is given in traveled distance per hour. Hyperactivity was defined as a significant increase in running activity, as determined by the analysis of running wheel activity within 24 h during the different phases.

For estrous cycle determination, vaginal smears were stained with 10% (v/v) Giemsa solution (Giemsa stock solution, ROTH T862.1, Karlsruhe, Germany). The vaginal smears were assigned to the different phases of the estrous cycle: estrous (fertile phase), metestrous, diestrous, and proestrous phase. The regular cycle of mice lasts 4 days, and if there is no fertile phase within 4 days, it is defined as amenorrhea.

2.3 Tissue Preparation and Brain Volume Measurement

The brains were sectioned frontally using a cryostat (Leica CM3050 S, Nussloch, Germany) into consecutive series of 40 µm thick sections. Every third section was thaw-mounted onto glass slides for subsequent brain volume analysis. For immunohistochemical studies, after the post-fixation step, brains were rinsed in tap water and went through baths of an ascending alcohol series, followed by xylol. Finally, they were embedded in paraffin and cut into 5 µm thick coronal slices.

For brain volume analysis, the sections were stained using Nissl standard protocols. The brain areas of stained slices were evaluated manually using ImageJ software (version 1.48v, Wayne Rasband, National Institutes of Health, Bethesda, MD, USA). An observer, blinded to the different experimental groups, manually traced every second slice. The volumes of interest were calculated using the Cavalieri method by multiplying individual areas with slice thickness, including the distance between the different slices, and summing the results. Using the Paxinos and Franklin Mouse Atlas [3], the cerebral cortex between Bregma 3.20 and -5.20, the hippocampus between Bregma -0.94 and -4.04, and the corpus callosum between Bregma 1.98 and -4.36, were included in the analyses.

The selection of these regions is based on the observation that patients with AN exhibit a significant reduction in the volume of grey and white matter, which is associated with neuropsychological deficits, including learning disabilities [4,5]. Specifically, a reduction in volume of the cerebral cortex, hippocampus, and corpus callosum has been observed in patients with AN [6–9].

2.5 Immunohistochemistry and Image Analysis

Epitope retrieval was performed by heat treatment in Tris/EDTA buffer (pH 9.0). Unspecific binding was blocked by incubation in blocking solution (5% normal goat serum in phosphate-buffered saline (PBS), pH 7.4) for 60 min. The primary antibody was diluted in blocking solution as specified in Supplementary Table 1, and sections were incubated overnight at 4 °C. After washing with PBS, secondary antibody incubation was carried out for 60 min. Labeling was visualized using 3,3’-diaminobenzidine (DAB; OriGene Technologies, Rockville, USA) for 10 min. Finally, all sections were dehydrated and mounted with Depex.

QuPath version 0.5.0 software [10] was used to quantify IBA1$^+$ cell densities. Cells were counted by two evaluators blinded to treatment groups and reported as mean cells per mm^2. All IBA1$^+$ cells with a visible soma were counted, excluding damaged sections.

Supplementary Table 1: Antibodies for immunohistochemistry.

Antigen	Species	Dilution	Purchase Number	RRID	Supplier
Primary Antibody					
IBA1	Rabbit polyclonal	1:750	019-19741	AB_839504	Wako, Osaka, Japan
Secondary Antibody					
EnVision+ Anti-rabbit	Goat	Ready to use	K4003	AB_2630375	Dako, Hamburg, Germany

For image analysis using Random Tree-based pixel classifiers, whole slide scans were performed using a Grundium Ocus 40 scanner (Grundium, Tampere, Finland). The classifier of QuPath was trained to exclude artifacts like dirt, folds, or unfocused areas from the ROI. The measurable area of the ROI corresponds to the total area minus the area classified as artifacts. For training, 8 randomly selected segments of all whole slide scans were used. The Random-Tree based pixel-classifier built into QuPath was then trained using manual annotations and providing all the available features at radii 1, 4 and 8 for the red, green and blue channel. Classification-quality was then checked subjectively for every evaluated ROI.

2.6 Morphological Analysis

For morphological analysis, cells at Bregma 0.14 were randomly selected by superimposing a rectangular grid with uniform distances between the lines in directions X and Y and analyzing all cells crossing the grid lines. Multifocal images (z-stack size: 0.1 µm) of the chosen cells were digitalized with the Leica DMC 6200 camera (Leica Microsystems CMS GmbH Wetzlar, Germany; 40-fold objective, NA: 0.95). The images were then imported into Neurolucida 360 where the cell bodies were reconstructed in the two-dimensional plane. The reconstruction of the cell processes were performed with the "smart-manual"-algorithm in centrifugal branch ordering under the consideration of their thickness. The digital cell models were later analyzed with the Neurolucida Explorer (Supplementary Video 1; MBF Bioscience, Willistion, USA; Version 2024.1.1). The following analysis options were chosen in batch analyses: cell bodies, segments, segment points and sholl analysis (Supplementary Figure 1; radius = 5 µm). The resulting data sets were then combined and organized using R (version 4.3.1 (2023-06-16 ucrt)).

2.7 Behavioral Tests

Before the start of each behavioral test, the mice were allowed to acclimatize in the test room for 30 min. Before feeding, the open field and novel object recognition tests were performed on half of the animals on one day and the other half on the following day. The forced swim test was performed on all animals on the first of the two test days after the feeding period, and the elevated plus maze test was performed on all animals on the following day after the feeding period.

For the forced swim test, the mice were placed in a glass cylinder (height and diameter = 20 cm) filled with water (height = 12 cm, 23-25 °C). Immobility was defined as floating with minimal movement, just enough to keep the nose above the water, while swimming involved visible paddling movements of

the fore or hind limbs. The times of swimming and immobile floating were measured manually with a stopwatch for 5 min under the observation of an experimenter and by recording with a camera. Mice that did not swim for longer than 30 sec were promptly withdrawn from the experiment to minimize stress levels. At the end of the test, the animals were dried with a towel and returned to their individual cages.

The elevated plus maze experimental set-up (65 cm x 65 cm) consists of a central platform (5 cm x 5 cm), two open and two closed arms (each 30 cm long) with Plexiglas walls (wall height = 15 cm, wall thickness = 0.5 cm). At the beginning of the test, a mouse was placed on the central platform. The time the animal spends in the open and closed arms was measured automatically via the VideoMot 2 analysis software (VideoMot 2, TSE Systems GmbH) during a 5 min test interval. For the OF test, the mouse was placed in an open box (50 cm x 50 cm) for 5 min. The time the mouse spent at the edge or in the center was automatically measured via the VideoMot 2 analysis software.

The novel object recognition test was conducted to determine whether the mouse was able to differentiate between a known and a novel object in a box. For acclimatization, the mice were placed individually in an open box, allowing them to explore it for 5 min (equals the OF test). After that, the animals were placed in the same box again, which contained two objects. During this training phase, both objects were identical, and the animals had 5 min to explore the objects. Objects of various geometric shapes (cylinder, cube, pyramid, star, 3-4 cm in height) made of wood coated with lacquer were used to encourage exploration, with positions and objects counterbalanced across groups and phases. Exploration was defined as the mouse being within 2 cm of the object, sniffing it, or touching it with its snout, but sitting on the object without active exploration was excluded. Following this, a one-hour break was implemented. One of the objects was substituted by a novel object, marking the recognition phase of the test. After that, the animals were reintroduced to the open field for 5 min. If the animal remembers the familiar object, it will show exploratory behavior towards the novel object due to curiosity. The box and all objects were disinfected with Bacillol® AF (propan-1-ol 45%, propan-2-ol 25%, ethanol 4.7%) after each trial. Finally, the exploration index was calculated as the ratio of the time spent exploring the novel object to the total time spent exploring both objects. An increased index indicates normal recognition memory.

2.8 Statistics

If the data did not follow a normal distribution, it was adjusted using the Box-Cox transformation with the most suitable λ value (serum NfL levels, hippocampal volume).

The statistical analyses were conducted with SPSS version 20 (IBM, Chicago, IL, USA) and GraphPad Prism 10.2 (GraphPad Software, Boston, MA, USA).

The target variable for sample size calculation was brain atrophy, specifically the volume of the cerebral cortex [11]. The volume of the SIH animals was reduced by 9% after starvation compared to the control group (SIH: 188.98 mm^3, SD: 5.07; control: 206.51 mm^3, SD: 3.61). This resulted in a Cohen's d effect size of 1.24, and a Cohen's f of 0.62 [12]. According to G-Power software, a total of 9 animals per group were required, but two additional animals were included to account for potential dropouts. Therefore, a total of 11 animals were used in the study, which is consistent with our previous study that used 11 SIH and 10 control animals.

Supplementary Table 2: All data and statistics.

A) AN-related symptoms

**Body weight**						
Phase	Group	N	Mean [g]	SD	p-value	Cohen's d
Acclimatization	Control	11	15.84	0.59	p = 0.17	0.64
	SIH	10	16.20	0.54		
Acute starvation	Control	11	16.95	0.38	p ≤ 0.001	-3.24
	SIH	10	13.49	1.50		
Chronic starvation	Control	11	18.60	0.61	p ≤ 0.001	-14.09
	SIH	10	12.16	0.17		
Refeeding	Control	11	20.23	0.49	p = 0.39	-0.39
	SIH	10	19.89	1.16		

**Running activity**						
Phase	Group	N	Mean [km]	SD	p-value	Cohen's d
Acclimatization	Control	11	4.79	0.82	p = 0.53	0.38
	SIH	10	5.14	0.99		
Acute starvation	Control	11	5.73	0.99	p ≤ 0.01	2.43
	SIH	10	9.08	1.71		
Chronic starvation	Control	11	4.93	1.02	p ≤ 0.001	3.41
	SIH	10	8.27	0.93		
Refeeding	Control	11	4.15	0.73	p ≤ 0.05	-0.99
	SIH	10	2.88	1.68		

**Incidence of estrous cycle**						
Block	Group	N	Incidence [%]	χ^2	p-value	Cohen's d
1	Control	11	45.5	2.65	p = 0.10	0.76
	SIH	10	80			
2	Control	11	90.9	0.96	p = 0.33	0.44
	SIH	10	100			
3	Control	11	63.6	4.49	p ≤ 0.05	1.04
	SIH	10	100			

4	Control	11	100	0	p = 1	0
	SIH	10	100			
5	Control	11	100	21	p ≤ 0.001	-∞
	SIH	10	0			
6	Control	11	90.9	17.36	p ≤ 0.001	-4.37
	SIH	10	0			
7	Control	11	100	21	p ≤ 0.001	-∞
	SIH	10	0			
8	Control	11	90.9	17.36	p ≤ 0.001	-4.37
	SIH	10	0			
9	Control	11	81.8	8.03	p ≤ 0.01	-1.57
	SIH	10	20			
10	Control	11	100	0	p = 1	0
	SIH	10	100			
11	Control	11	72.7	0.15	p = 0.70	0.17
	SIH	10	80			
12	Control	11	100	1.16	p = 0.28	0.48
	SIH	10	90			
13	Control	11	81.8	2.01	p = 0.16	0.65
	SIH	10	100			

B) Serum NfL levels

Phase	Group	N	Mean [pg/ml]	SD	p-value	Cohen's d
Chronic starvation	Control	9	28.49	15.89	p ≤ 0.001	1.88
	SIH	8	72.00	28.56		
Refeeding	Control	9	40.17	15.35	p = 0.47	-1.20
	SIH	7	25.47	6.04		

C) Brain volumes

Cerebral cortex volume						
Phase	Group	N	Mean [mm^3]	SD	p-value	Cohen's d
Chronic starvation	Control	5	46.17	0.77	p ≤ 0.001	-2.96
	SIH	9	40.00	2.49		
Refeeding	Control	11	45.84	2.11	p > 0.99	-0.03
	SIH	10	45.78	2.50		

Hippocampal volume						
Phase	Group	N	Mean [mm^3]	SD	p-value	Cohen's d
Chronic starvation	Control	5	11.14	0.40	p ≤ 0.01	-2.28
	SIH	9	10.11	0.48		
Refeeding	Control	11	10.96	0.78	p = 0.37	0.54
	SIH	10	11.30	0.39		

Corpus callosum volume						
Phase	Group	N	Mean [mm^3]	SD	p-value	Cohen's d
Chronic starvation	Control	5	3.91	0.17	p ≤ 0.001	-2.48
	SIH	9	3.41	0.21		
Refeeding	Control	11	4.56	0.12	p ≤ 0.01	-1.55
	SIH	10	4.28	0.23		

D) Behavioral tests

Forced swim test						
Phase	Group	N	Mean [%]	SD	p-value	Cohen's d
Acclimatization	Control	11	50.70	16.65	p = 0.94	0.29
	SIH	11	54.58	8.81		
Acute starvation	Control	11	73.24	17.54	p = 0.97	0.24
	SIH	10	76.57	8.01		
Chronic starvation	Control	11	70.39	17.49	p = 0.75	-0.46
	SIH	10	63.40	11.86		

Refeeding	Control	11	62.79	16.48	p = 0.33	0.76
	SIH	10	72.20	4.77		

**Elevated plus maze test**						
Phase	Group	N	Mean [%]	SD	p-value	Cohen's d
Acclimatization	Control	10	8.52	6.24	p > 0.99	0.15
	SIH	11	9.64	8.14		
Acute starvation	Control	11	7.83	3.50	p = 0.83	0.43
	SIH	10	10.18	6.99		
Chronic starvation	Control	11	3.80	2.55	p ≤ 0.05	1.48
	SIH	10	12.99	8.64		
Refeeding	Control	11	1.72	2.00	p = 0.65	0.54
	SIH	10	2.89	2.32		

**Open field test**						
Phase	Group	N	Mean [%]	SD	p-value	Cohen's d
Acclimatization	Control	11	10.89	4.45	p > 0.99	-0.13
	SIH	11	10.36	4.04		
Acute starvation	Control	11	7.04	3.56	p > 0.99	0.43
	SIH	11	8.96	5.18		
Chronic starvation	Control	11	7.01	5.29	p > 0.99	0.40
	SIH	10	9.25	5.95		
Refeeding	Control	11	8.65	8.47	p = 0.29	0.59
	SIH	10	13.19	7.00		

**Novel object recognition test**						
Phase	Group	N	Mean	SD	p-value	Cohen's d
Acclimatization	Control	11	0.47	0.11	p > 0.99	-0.31
	SIH	10	0.43	0.16		
Acute starvation	Control	9	0.38	0.12	p = 0.66	0.77
	SIH	8	0.55	0.30		

Chronic starvation	Control	11	0.51	0.13	p > 0.99	-0.21
	SIH	9	0.46	0.29		
Refeeding	Control	10	0.45	0.12	p ≤ 0.05	1.53
	SIH	10	0.61	0.09		

E) IBA1 staining

Cell density						
Phase	Group	N	Mean [cells/mm²]	SD	p-value	Cohen's d
Chronic starvation	Control	4	78.27	6.82	p ≤ 0.05	-2.08
	SIH	5	63.02	7.67		

Soma area to total area						
Phase	Group	N	Mean [%]	SD	p-value	Cohen's d
Chronic starvation	Control	4	0.76	0.12	p ≤ 0.01	-2.71
	SIH	5	0.45	0.11		

Process area to total area						
Phase	Group	N	Mean [%]	SD	p-value	Cohen's d
Chronic starvation	Control	4	8.90	0.90	p ≤ 0.001	-5.64
	SIH	5	4.88	0.53		

Ratio process/soma area						
Phase	Group	N	Mean	SD	p-value	Cohen's d
Chronic starvation	Control	4	12.51	1.32	p = 0.41	-0.59
	SIH	5	11.46	2.08		

F) Morphological analysis

Soma area per cell						
Phase	Group	N [cells]	Mean [μm^2]	SD	p-value	Cohen's d
Chronic starvation	Control	89	48.76	14.55	p ≤ 0.05	0.48
	SIH	65	55.81	15.21		

Processes per cell						
Phase	Group	N [cells]	Mean [n]	SD	p-value	Cohen's d
Chronic starvation	Control	89	6.07	2.21	p = 0.76	-0.15
	SIH	65	5.75	1.91		

Mean process length						
Phase	Group	N [cells]	Mean [µm]	SD	p-value	Cohen's d
Chronic starvation	Control	87	39.60	19.72	$p \leq 0.05$	0.29
	SIH	65	48.98	26.00		

Nodes per cell						
Phase	Group	N [cells]	Mean [n]	SD	p-value	Cohen's d
Chronic starvation	Control	89	31.37	17.47	p = 0.19	0.15
	SIH	64	34.11	18.10		

Endpoints per cell						
Phase	Group	N [cells]	Mean [n]	SD	p-value	Cohen's d
Chronic starvation	Control	89	41.10	20.35	p = 0.12	0.20
	SIH	64	45.19	21.20		

Ramification index						
Phase	Group	N [cells]	Mean	SD	p-value	Cohen's d
Chronic starvation	Control	88	0.222	0.068	$p \leq 0.1$	0.20
	SIH	65	0.241	0.078		

Sholl analysis - intersections						
Phase	Group	N [cells]	Mean [n]	SD	p-value	Cohen's d
Chronic starvation	Control	89	2.58	4.00	p = 0.14	0.08
	SIH	65	2.92	4.64		

Sholl shell	Group	N [cells]	Mean	SD	p-value	Cohen's d
5 µm	Control	89	8.10	4.47	p = 0.08	-0.19
	SIH	65	7.28	4.44		
10 µm	Control	89	7.67	4.03	p ≤ 0.001	0.57
	SIH	65	10.37	5.26		
15 µm	Control	89	5.98	4.29	p ≤ 0.001	0.31
	SIH	65	7.57	5.69		
20 µm	Control	89	4.26	4.03	p = 0.13	0.17
	SIH	65	4.95	4.08		
25 µm	Control	89	2.33	2.49	p = 0.80	0.05
	SIH	65	2.45	2.71		
30 µm	Control	89	1.32	2.15	p = 0.93	0.02
	SIH	65	1.35	2.22		
35 µm	Control	89	0.78	1.86	p = 0.73	-0.10
	SIH	65	0.62	1.26		
40 µm	Control	89	0.36	1.16	p = 0.78	-0.13
	SIH	65	0.23	0.68		
45 µm	Control	89	0.25	0.92	p = 0.84	-0.11
	SIH	65	0.15	0.83		
50 µm	Control	89	0.022	0.21	p = 0.99	-0.04
	SIH	65	0.015	0.12		
55 µm	Control	89	0	0	p = 0.97	0.19
	SIH	65	0.015	0.12		
60 µm	Control	89	0	0	p = 1	-
	SIH	65	0	0		

Sholl analysis - volume of processes						
Phase	Group	N [cells]	Mean [µm^3]	SD	p-value	Cohen's d
Chronic starvation	Control	89	5.9	9.43	p ≤ 0.001	0.17
	SIH	65	7.81	13.04		

Sholl shell	Group	N [cells]	Mean [µm^3]	SD	p-value	Cohen's d
5 µm	Control	89	5.45	5.34	p > 0.99	0.02
	SIH	65	5.58	6.46		
10 µm	Control	89	20.96	12.60	p ≤ 0.01	0.64
	SIH	65	30.14	16.50		
15 µm	Control	89	14.62	10.56	p ≤ 0.01	0.68
	SIH	65	23.37	15.48		
20 µm	Control	89	10.25	10.12	p ≤ 0.05	0.54
	SIH	65	16.63	13.78		
25 µm	Control	89	6.01	6.54	p = 0.41	0.38
	SIH	65	9.0	9.31		
30 µm	Control	89	3.73	5.47	p > 0.99	0.19
	SIH	65	4.91	6.91		
35 µm	Control	89	2.22	5.87	p > 0.99	0.04
	SIH	65	2.44	4.19		
40 µm	Control	89	1.02	3.10	p > 0.99	0.02
	SIH	65	1.08	3.06		
45 µm	Control	89	0.46	1.68	p > 0.99	-0.01
	SIH	65	0.44	1.90		
50 µm	Control	89	0.12	0.49	p > 0.99	-0.03
	SIH	65	0.11	0.63		
55 µm	Control	89	0.005	0.05	p > 0.99	0.14
	SIH	65	0.022	0.18		
60 µm	Control	89	0	0	p > 0.99	0.19
	SIH	65	0.033	0.26		

Supplementary Video 1

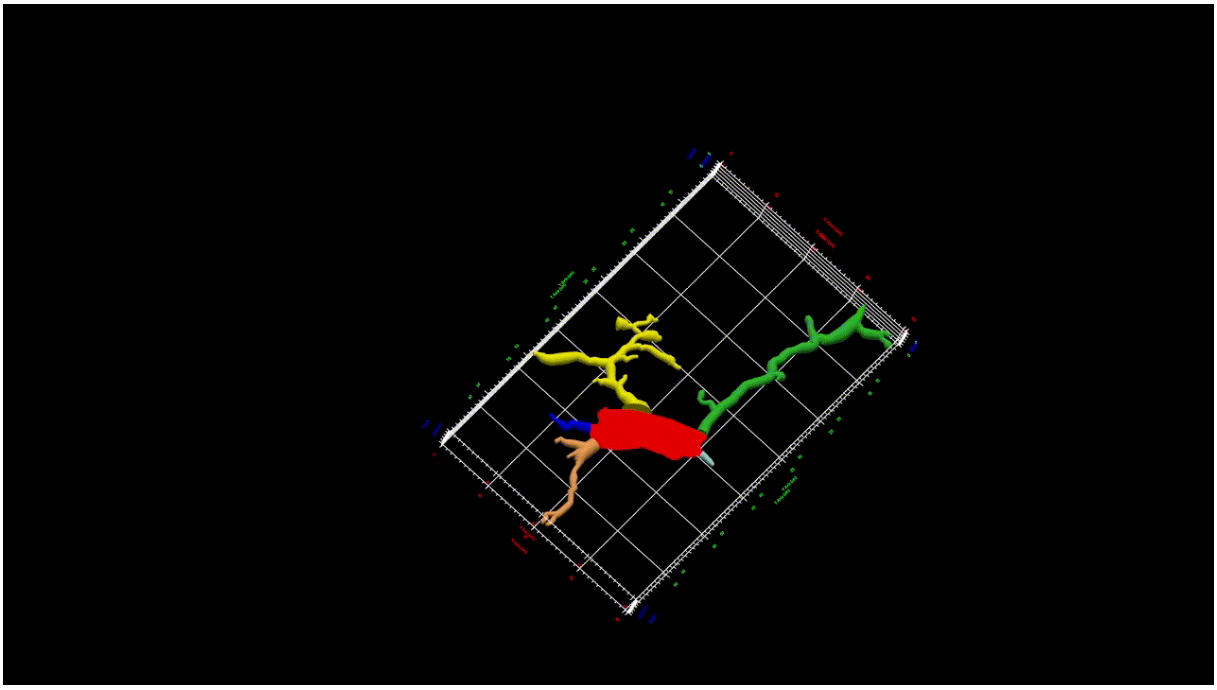

Video S1: Rotating SIH microglial cell in a three-dimensional environment reconstructed in Neurolucida 360.

Supplementary Figure 1

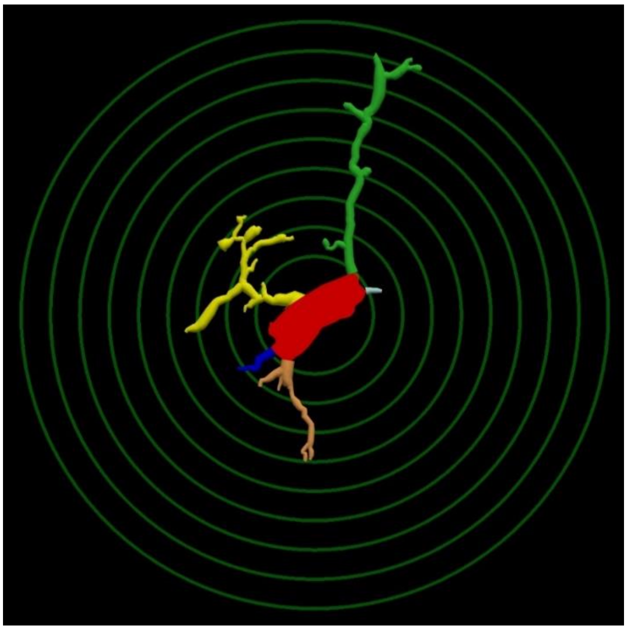

Figure S1: Sholl analysis (radius = 5 µm) performed on a SIH microglial cell reconstructed in Neurolucida 360.

References

1. Gabloffsky, T.; Gill, S.; Staffeld, A.; Salomon, R.; Power Guerra, N.; Joost, S.; Hawlitschka, A.; Kipp, M.; Frintrop, L. Food Restriction in Mice Induces Food-Anticipatory Activity and Circadian-Rhythm-Related Activity Changes. *Nutrients* **2022**, *14*, doi:10.3390/nu14245252.
2. Staffeld, A.; Gill, S.; Zimmermann, A.; Böge, N.; Schuster, K.; Lang, S.; Kipp, M.; Palme, R.; Frintrop, L. Establishment of a Murine Chronic Anorexia Nervosa Model. *Cells* **2023**, *12*, doi:10.3390/cells12131710.
3. Franklin, K.B.J.; Paxinos, G. *The mouse brain in stereotaxic coordinates*, 2. ed., [Nachdr.]; Acad. Press: San Diego, Calif., 2005, ISBN 012547637X.
4. Castro-Fornieles, J.; Caldú, X.; Andrés-Perpiñá, S.; Lázaro, L.; Bargalló, N.; Falcón, C.; Plana, M.T.; Junqué, C. A cross-sectional and follow-up functional MRI study with a working memory task in adolescent anorexia nervosa. *Neuropsychologia* **2010**, *48*, 4111–4116, doi:10.1016/j.neuropsychologia.2010.10.003.
5. McCormick, L.M.; Keel, P.K.; Brumm, M.C.; Bowers, W.; Swayze, V.; Andersen, A.; Andreasen, N. Implications of starvation-induced change in right dorsal anterior cingulate volume in anorexia nervosa. *Int. J. Eat. Disord.* **2008**, *41*, 602–610, doi:10.1002/eat.20549.
6. Tose, K.; Takamura, T.; Isobe, M.; Hirano, Y.; Sato, Y.; Kodama, N.; Yoshihara, K.; Maikusa, N.; Moriguchi, Y.; Noda, T.; et al. Systematic reduction of gray matter volume in anorexia nervosa, but relative enlargement with clinical symptoms in the prefrontal and posterior insular cortices: a multicenter neuroimaging study. *Mol. Psychiatry* **2024**, *29*, 891–901, doi:10.1038/s41380-023-02378-4.
7. Walton, E.; Bernardoni, F.; Batury, V.-L.; Bahnsen, K.; Larivière, S.; Abbate-Daga, G.; Andres-Perpiña, S.; Bang, L.; Bischoff-Grethe, A.; Brooks, S.J.; et al. Brain Structure in Acutely Underweight and Partially Weight-Restored Individuals With Anorexia Nervosa: A Coordinated Analysis by the ENIGMA Eating Disorders Working Group. *Biol. Psychiatry* **2022**, *92*, 730–738, doi:10.1016/j.biopsych.2022.04.022.
8. Seitz, J.; Bühren, K.; Polier, G.G. von; Heussen, N.; Herpertz-Dahlmann, B.; Konrad, K. Morphological changes in the brain of acutely ill and weight-recovered patients with anorexia nervosa. A meta-analysis and qualitative review. *Z. Kinder Jugendpsychiatr. Psychother.* **2014**, *42*, 7-17; quiz 17-8, doi:10.1024/1422-4917/a000265.
9. Bahnsen, K.; Wronski, M.-L.; Keeler, J.L.; King, J.A.; Preusker, Q.; Kolb, T.; Weidner, K.; Roessner, V.; Bernardoni, F.; Ehrlich, S. Differential longitudinal changes of hippocampal subfields in patients with anorexia nervosa. *Psychiatry Clin. Neurosci.* **2024**, *78*, 186–196, doi:10.1111/pcn.13626.
10. Bankhead, P.; Loughrey, M.B.; Fernández, J.A.; Dombrowski, Y.; McArt, D.G.; Dunne, P.D.; McQuaid, S.; Gray, R.T.; Murray, L.J.; Coleman, H.G.; et al. QuPath: Open source software for digital pathology image analysis. *Sci. Rep.* **2017**, *7*, 16878, doi:10.1038/s41598-017-17204-5.
11. Frintrop, L.; Trinh, S.; Liesbrock, J.; Leunissen, C.; Kempermann, J.; Etdöger, S.; Kas, M.J.; Tolba, R.; Heussen, N.; Neulen, J.; et al. The reduction of astrocytes and brain volume loss in anorexia nervosa-the impact of starvation and refeeding in a rodent model. *Transl. Psychiatry* **2019**, *9*, 159, doi:10.1038/s41398-019-0493-7.
12. Wolfgang Lenhard; Alexandra Lenhard. *Computation of Effect Sizes*, 2017.
