# Supplementary figures and images for: Increased Serum Neurofilament Light Chain Concentration Associated With Microglial Morphology Changes in Chronically‐Starved Mice
Source: Int J Eat Disord. 2025 Mar 22;58(6):1130–43. doi: 10.1002/eat.24423 (PMC12138848; doi:10.1002/eat.24423)

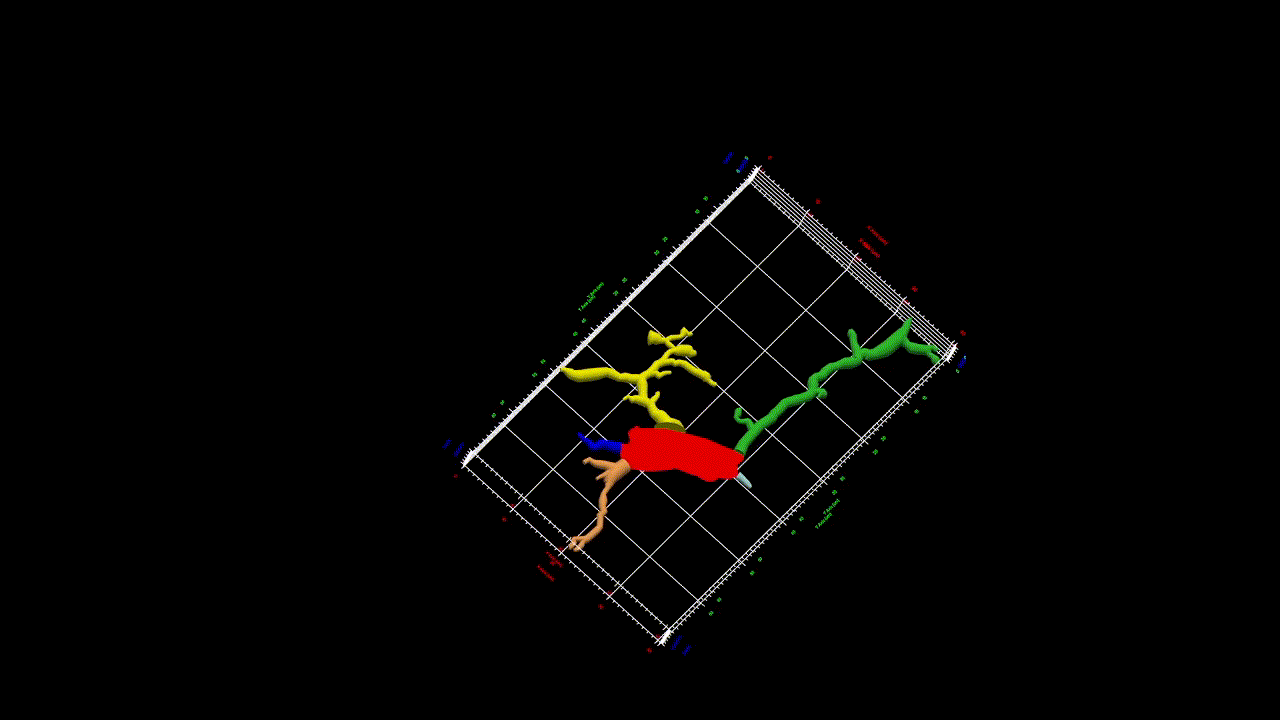

Supplement: Supplementary file 2 — Video S1. [file EAT-58-1130-s002.gif]
